# Determinants of excessive gestational weight gain: a systematic review and meta-analysis

**DOI:** 10.1186/s13690-022-00864-9

**Published:** 2022-05-03

**Authors:** Meng Zhou, Xueqing Peng, Honggang Yi, Shaowen Tang, Hua You

**Affiliations:** Nanjing Medical University, Nanjing, Jiangsu China

**Keywords:** excessive gestational weight gain, Determinants, systematic review, meta-analysis

## Abstract

**Background:**

The prevalence of excessive gestational weight gain (EGWG) during pregnancy is increasing, and it is extremely harmful to pregnant women and newborns. Previous studies have suggested that EGWG is associated with various factors. We conducted a systematic review and meta-analysis to identify, quantify and analyze determinants of EGWG and evaluate the effect of these determinants on EGWG.

**Methods:**

We searched for articles, from January 2009 to November 2020, related to the determinants of EGWG during pregnancy using four Chinese and four English databases. The Preferred Reporting Items for Systematic Reviews and Meta-analysis (PRISMA) statement was utilized to guide the systematic review and meta-analysis process.

**Results:**

Seventy studies, which identified EGWG factors in pregnant women (58 factors, 3 themes: individual [7 aspects, 37 factors]; family [4 aspects, 8 factors]; and social [4 aspects, 13 factors]), were included and analyzed in the systematic review. A meta-analysis was conducted for 13 factors (including 10 individual factors, 2 family factors, and 1 social factor) and revealed that pre-pregnancy overweight (including obesity), younger age (≤ 30 years old), unemployed, primiparity, smoking, and being unmarried (including divorced) were risk factors for EGWG, while prepregnancy underweight and inadequate antenatal care were protective factors for EGWG. There was no significant correlation between EGWG and education level, alcohol consumption, planning pregnancy, food security, and whether access to nutrition guidance during pregnancy.

**Conclusions:**

EGWG was prevalent in pregnant women, and its prevalence seemed to be high and similar in many countries. Based on observational studies with medium-level and high-level evidence, some individual, family, and social factors were found to be associated with EGWG using qualitative and quantitative methods. In the future, exposure of pregnant women to risk factors for EGWG should be avoided, and interventions should be developed around the identified factors.

**Supplementary Information:**

The online version contains supplementary material available at 10.1186/s13690-022-00864-9.

## Background

Excessive gestational weight gain (EGWG) is becoming a worldwide epidemic. Multiple studies conducted in different regions of the world have recorded the prevalence of EGWG in pregnant women. According to previous studies, more than half of pregnant women in the United States had EGWG [1]. The EGWG rate of Chinese pregnant women is between 46.5% and 53.6% in China [2]. Studies have showed that pregnant women with overweight or obesity are more likely to develop EGWG [3]. There are high rates of overweight and obesity among pregnant women worldwide. For example, 40% of pregnant women in England are overweight or obese [4]. In Australia, 13% of pregnant women are overweight, and 20% are obese [5]. This also indirectly reflects the possibility of a high prevalence of EGWG. The problem of EGWG should be seriously considered.

To alleviate the increasingly serious problem among these special groups, the Institute of Medicine (IOM) revised its guidelines for weight gain during pregnancy in 2009. Compared to the guidelines proposed in 1990, the 2009 version focused more on healthy pregnancy outcomes, postpartum weight retention, and childhood obesity. According to IOM guidelines, the recommended weight gains for underweight, normal weight, overweight and obese women were 12.5–18, 11.5–16, 7.0–11.5, and 5.0–9.0 kg, respectively [6]. Pregnancy weight gain (kg) is equal to the weight before delivery (kg) minus the weight before pregnancy (kg). Gestational weight gain beyond the recommended levels of the IOM guideline is defined as EGWG [6].

Several studies have reported that EGWG in pregnant women could increase the risk of adverse pregnancy outcomes for both mothers and their offspring [2]. For mothers, adverse outcomes included postpartum hemorrhage, postpartum weight retention, and premature termination of breast milk after delivery. Moreover, excessive weight gain was associated with cesarean delivery, prolonged second stage of delivery, and spontaneous preterm birth [5, 7]. In addition to being large at birth, infants born to mothers with EGWG were more likely to have meconium inhalation syndrome, cephalopelvic disproportion, shoulder dystocia, and asphyxia [8]. It also enhanced the risk of complications at multiple of life, such as childhood obesity, diabetes, cardiovascular disease, cancer, and metabolic syndrome [6].

EGWG is a complex clinical picture associated with physical, physiological, hormonal, genetic, cultural, socioeconomic, and environmental factors [6]. Although many studies have explored the determinants of EGWG, they had several limitations, such as small study population, the differences in sample size, scattered determinants, and inconsistent results. For example, several studies reported that older pregnant women were at lower risk for EGWG [9–12], but some studies have reported the opposite results [13, 14]. Therefore, the purpose of this study was to identify, quantify and analyze the determinants of EGWG by conducting a systematic review and meta-analysis. Our findings would be beneficial to formulate scientific intervention measures for pregnancy weight management based on relevant factors, provide a reference for the government to issue pregnancy weight management policies.

## Methods

This study was conducted according to the Preferred Reporting Items for Systematic Reviews and Meta-analysis (PRISMA) 2020 statement [15]. The review protocol was registered in the International Prospective Register of Systematic Reviews (www.crd.york.ac.uk/PROSPERO CRD42021229694).

### Search strategy and study selection

The research question was defined by the population, intervention, comparison, outcome (PICO) model following the PRISMA statement [15]: Population, the pregnant women; Intervention, being exposed to determinants for EGWG; Comparison, not being exposed to determinants for EGWG; Outcomes, EGWG.

According to the search scheme established in the PROSPERO review protocol, we searched the following electronic bibliographic databases: PubMed, Web of Science (WOS), Cochrane Library (Trails), Embase, China National Knowledge Internet Database (CNKI), Wanfang Data, the VIP Database for Chinese Technical Periodicals (VIP), and the Chinese BioMedical Literature Database (SinoMed). The search strategy was based on a combination of the following terms: [(gestational weight gain) OR (excessive gestational weight gain) OR (maternal obesity) OR (maternal overweight)] AND [(risk factor*) OR (association factor*) OR (influencing factor*) OR (determinant*)]. The complete search strategy is provided in Additional File 1.

Two independent reviewers screened the studies according to the following inclusion criteria at the same time. Disagreements were resolved through discussion between the two reviewers and full group consensus. Otherwise, the references of the included studies were also tracked for more suitable studies. The inclusion criteria were as follows: (1) studies published between January 2009 and November 30, 2020; (2) the study was related to the determinants of EGWG in pregnant women, including individual, family, social and other factors. (3) the participants were singleton pregnant women with no primary pregnancy-related diseases before pregnancy; (4) the study was a cohort study, case–control study, or cross-sectional study; (5) The definition of EGWG followed the IOM guidelines revised in 2009. (6) for the research on the same sample (i.e., academic dissertation and journal articles), we selected paper with higher quality to be included; and (7) the study was published in Chinese or English.

### Assessment of quality of evidence

Two reviewers independently completed the quality assessment of the literature. Instances in which the two raters disagreed were reviewed by senior study personnel to determine the reported quality ratings.

The Newcastle − Ottawa Scale (NOS) was used to evaluate the risk of bias in cohort studies and case–control studies from three aspects: study population selection (4 items), comparability between groups (1 item), and measurement of outcomes (3 items). The total score of the above items was 9 points. All eligible cross-sectional studies were examined using 11 items recommended by the Agency for Healthcare Research and Quality (AHRQ), including data source, sample representativeness, a detailed description of the object and research methods, and identification of confounding factors. The overall risk of bias was determined a priori and judged as follows: a score of 0 − 3 was considered low quality, 4 − 6 was medium quality and > 7 was high-quality [16].

### Data extraction

Data extraction was conducted by two reviewers independently and discussed with the third senior study personnel if remained disagreement, followed four steps: (1) A standard template was designed for data extraction of study and sample characteristics, including general characteristics (title, author, year, region), research methods (study design, sample size, EGWG occurrence numbers), determinants, and key findings. (2) Odds ratio (OR) or risk ratio (RR) —two reviewers extracted data corresponding to each factor, including factor definition, correlation strength index OR, RR, 95% confidence interval (CI), analysis method (univariate analysis or multivariate analysis), and adjustment variables. Only the studies, which were provided OR/ RR values directly or calculated OR/RR values based on the study data, were included. If there was a univariate or multivariate analysis, the multivariate results were considered first. Analysis results after the adjustment for covariables were preferred. If the OR/RR values could not be extracted directly from the original text, we extracted the original data. The OR value of the determinant was then calculated using the original data. (3) Data conversion: according to the most common classification methods in the included studies or the existing classification standards of the study itself, the data of multiclassification were converted into binary classification data (i.e., Age [≤ 30 *vs* > 30] and education level [low *vs*. high]). (4) Data merge—two methods were used to obtain the OR value of the binary data merge. One was based on the original data, and the other combined the OR value of multiple categories using RevMan 5.3 software. For example, since age is multiclassification data, all categories (≤ 30 years old) were combined in one study at first. We then calculated the OR value of this determinant (≤ 30 years old) for EGWG. Lastly, the OR values were combined in all studies that mentioned this factor.

### Data analysis

In the meta-analysis, the OR value was used to calculate the combined effect size and its 95% CI, which was calculated from the original data or mentioned in the original text (RR value approximately equal to OR value). The heterogeneity among the studies was evaluated using the Q test combined with I^2^. If *P* ≥ 0. 05 and I^2^ < 50%, a fixed-effects model was selected for the meta-analysis. If *P* < 0.05 and I^2^ ≥ 50%, the DerSimonian-Laird random-effects model was used to obtain the combined OR and 95%CI for the determinants.

Sensitivity analysis was performed first using the one-by-one exclusion method, and then single-factor subgroup analysis and meta-regression were performed (i.e., year, region, and study type). In addition, regions were divided after considering economic differences. The division of years was based on a similar number of studies conducted around 2015. Funnel plots and Egger’s test were being used to evaluate and judge the publication bias of the factors mentioned in more than 10 studies. If the points on both sides of the funnel plot were funneled in the middle or approximately symmetrical, there was no obvious publication bias. The *P*-value of Egger's test (< 0.05) was considered an obvious publication bias.

In this study, RevMan 5.3 and Stata 16.0 were used for the above statistical analysis, and the test level was set at *P* < 0.05. The prevalence of consolidation and 95% CI were calculated using the total number of participants and the total number of EGWG occurrences for cohort studies using OpenMeta software [17].

This study was based on the framework of the social determinants of health (SDH), which were proposed by the 66^th^ World Health Assembly of the World Health Organization (WHO); the determinants of EGWG were categorized into three themes—individual, family, and social factors [18]. Considering all factors synthetically, quantitative analysis was carried out for the factors discussed in more than three studies, and other factors were described qualitatively. The meta-analysis was conducted for 10 individual factors, 2 family factors, and 1 social factor.

## Results

**Fig. 1:**
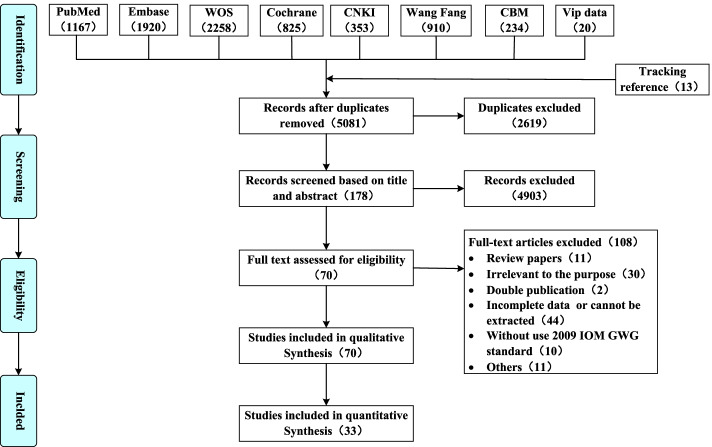
Flow diagram of study selection

The selection process is illustrated in the flow diagram shown in Fig. 1. The database search returned 7,700 records. After removing the duplicates, 5,081 articles were screened for eligibility. A total of 4,903 studies were excluded based on title and abstract, and 108 were excluded based on full-text screening, resulting in 57 studies being included. In addition, we tracked the article references and added 13 articles. Finally, 70 studies were included in the qualitative synthesis and 33 in the quantitative synthesis.

### Basic characteristics of the included studies

The basic information for each included study is shown in Additional File 2. Seventy studies were published from January 2009 to November 2020, including 48 cohort studies (68.6%), 7 case–control studies (10.0%), and 15 cross-sectional studies (21.4%); 10% of them were in Chinese (*N* = 7) and the rest were in English. The included studies were conducted in Americas (*n* = 33), Asia (*n* = 22), Europe (*n* = 11), Oceania (*n* = 3), and Africa (*n* = 1).

In the 48 cohort studies, 3,053,581 women were investigated worldwide, 1,473,843 of them had EGWG, and the prevalence of EGWG was approximately 45.5% (95% CI:42.8%, 48.3%). Among the different regions, the prevalence of EGWG was highest in Americas, 49.8% (95% CI, 46.9% − 52.8%) and Africa (55.20%), followed by Oceania at 49.0% (95% CI, 17.9% − 80.2%) and Europe (42.3% 95% CI:35.9% − 48.6%), and the lowest prevalence in Asia at 38.2% (95% CI, 27.8% − 48.6%).

### Quality of the literature

For full quality appraisal, see Additional File 3. The quality of all included studies was medium to high, with scores of > 4 points. Thirty-eight articles (54.3%) scored more than 7 points and 32 articles were rated as medium quality, suggesting that the overall quality of the included studies was acceptable.

### The determinants for EGWG

**Fig. 2:**
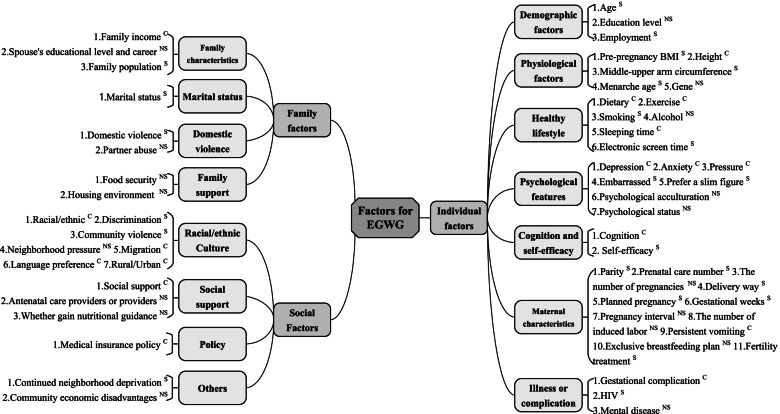
Classification of influencing factors for EGWG

  According to the conceptual framework of the SDH, all factors were identified (58 factors, 3 themes—individual [7 aspects, 37 factors]; family [4 aspects, 8 factors]; and social [4 aspects, 13 factors] (Fig. 2). Therefore, a meta-analysis was conducted for 13 factors (including 10 individual factors, 2 family factors, and 1 social factor) (Table 1). The results were statistically concluded as six risk factors and two protective factors for EGWG.

**Table 1:** Meta-analysis of risk factors for excessive gestational weight gain

Risk factors	Studies (n)	Samples (n)	Effects model	OR	95%CI		*P* value	I^2^	Study ID^a^
					**CL**	**CU**			
**Individual factors**									
Age, years (< 30 *vs.* ≥ 30)	13	65,528	Fixed	1.14	1.10	1.19	< 0.01	41%	[2, 3, 5, 10, 20, 25-27, 29, 46, 50, 59, 61]
Employment (No *vs.* Yes)	9	57,241	Fixed	1.07	1.02	1.12	< 0.01	12%	[15, 16, 20, 21, 25-27, 29, 59]
Education level (Low *vs.* High)	18	294,934	Random	1.06	0.92	1.21	0.44	93%	[1, 2, 5, 14-17, 20, 21, 25-27, 32, 40, 49, 50, 61, 66]
Pregnancy BMI, kg/m^2^ (Underweight *vs.* Normal)	21	342,606	Fixed	0.56	0.54	0.59	< 0.01	48%	[2, 3, 5, 8, 9, 11, 14, 15, 17, 25-27, 40, 46, 49-51, 58, 59, 64, 66]
Pregnancy BMI (kg/m^2^) (Overweight, obese *vs.* Normal)	23	343,652	Random	2.49	2.11	2.94	< 0.01	97%	[2, 3, 5, 8, 9, 11, 14, 15, 17, 25-27, 29, 40, 46, 49-51, 58, 59, 61, 64, 66]
Parity (Primiparity *vs.* Multiparity)	18	294,185	Fixed	1.30	1.28	1.32	< 0.01	38%	[1, 2, 5, 9, 15, 20, 21, 25-27, 29, 32, 40, 46, 50, 59, 61, 66]
Planned pregnancy (No *vs.* Yes)	3	76,056	Fixed	0.92	0.79	1.07	0.280	0%	[16, 26, 47]
Smoking (Yes *vs.* No)	12	237,046	Fixed	1.29	1.25	1.34	< 0.01	13%	[2, 14, 21, 25, 26, 32, 40, 46, 49, 59, 61, 66]
Alcohol (Yes *vs.* No)	6	233,736	Random	1.90	0.50	7.22	0.34	99%	[2, 25, 26, 40, 49, 59]
Prenatal care (Inadequate *vs.* Adequate)	5	232,597	Random	0.68	0.55	0.84	< 0.01	62%	[15-17, 40, 49]
**Family factors**									
Marital status (Unmarried, divorced *vs.* Married)	9	287,737	Fixed	1.20	1.18	1.23	< 0.01	0%	[15, 25-27, 29, 40, 59, 61, 66]
Food security (No *vs.* Yes)	4	1794	Fixed	1.13	0.84	1.5	0.42	0%	[16, 21, 29, 32]
**Social factors**									
Nutrition advice or guidance (Not have *vs.* Have)	3	1575	Fixed	1.08	0.88	1.32	0.47	0%	[17, 29, 49]

^a^The reference details correspond to study ID are shown in Additional file 2

### Individual factors

#### Demographic factors

Six studies showed a lower risk of EGWG in older pregnant women [9–12], but two studies demonstrated the opposite results [13, 14], and the remaining studies did not show a significant association between EGWG and age. The age of 30 years was used as the classification basis according to the most common classification method in the included studies. Meta-analysis showed that the younger age (≤ 30 years) was a risk factor for EGWG (OR, 1.14; 95% CI, 1.10 − 1.19) (Table 1).

According to the division of educational level and the years of education mentioned in the original text, the educational level was divided into high and low levels. Significant heterogeneity was observed in the fixed-effects model (I^2^ = 93%; *P* < 0.01). After adjusting to the random-effects model, the results demonstrated that there was no correlation (OR, 1.06; 95% CI, 0.92 − 1.21; *P* = 0.44) (Table 1).

The employment status of pregnant women was not associated with EGWG [10, 19–26], but the meta-analysis found that unemployed pregnant women were more likely to develop EGWG (OR, 1.07; 95% CI, 1.02 − 1.12) (Table 1).

#### Physiological and anthropometric factors

Based on the classification methods mentioned in the literature, the prepregnancy body mass index (BMI) was divided into overweight (including obesity), normal weight and underweight. Because the number of pregnant women with obesity was relatively small, the obese group and the overweight groups were combined. Women with prepregnancy overweight (including obesity) were more likely to develop EGWG at normal weight (OR, 2.49; 95% CI, 2.11 − 2.94) and women with prepregnancy underweight had about half the odds of developing EGWG (OR, 0.56; 95% CI, 0.54 − 0.59) (Table 1).

#### Healthy lifestyle

In terms of diet, unreasonable energy intake was a risk factor for EGWG [13, 23, 27–29]. A high-calorie diet, such as the Western diet model [30]and high-carbohydrate low-fat intake diet was the risk factor [31]. Higher healthy dietary scores also improved the risk of EGWG [28]. Some dietary patterns were protective factors, such as those based on grains, vegetables, legumes, marine fish, milk, and dairy products and the avoidance of snacks between meals [32, 33]. However, excessive intake of vegetables, fruits, fish, and seafood can increase the risk of EGWG [9, 23, 34].

In terms of exercise, pregnant women who exercised less had a higher chance of EGWG [2, 9, 35]. Regular physical exercise reduced the risk of EGWG in pregnant women [21, 24, 36, 37]. However, one study showed the opposite result [11].

Smoking status was classified as smoking (including quitting) and nonsmoking to perform a meta-analysis, and the probability of EGWG in pregnant women with smoking was 1.29 times higher than in those without smoking (OR, 1.29; 95% CI, 1.25 − 1.34) (Table 1). Particularly, women exposed to passive smoking in the third trimester had a reduced risk of EGWG [38].

One study reported that pregnant women who consumed alcohol during pregnancy were at low risk of EGWG [29], but most studies reported the opposite results [22, 23, 26, 36, 39–41], consistent with the findings of the meta-analysis (OR, 1.90; 95% CI, 0.50 − 7.22) (Table 1).

#### Psychological feature

Two studies reported that psychological distress during pregnancy, such as depression [23] and anxiety [42], reduced the risk of EGWG. However, other studies reported that psychological distress was not associated with EGWG [24, 36, 43–46]. Pregnancy stress had no effect on EGWG [23, 36, 38, 44, 45], while depression, stress, anxiety, and pregnancy-related anxiety had positive effects on gestational weight gain, although the specific influence on EGWG was unclear [20].

#### Cognition and self-efficacy

Pregnant women with lower self-efficacy in weight management and cognitive level of risk factors for infants were at risk of EGWG [40, 45].

#### Maternal characteristics

Most studies mentioned the relationship between parity and EGWG, 11 of which reported that primipara was more likely to develop EGWG [10, 21, 23, 29, 36, 39, 40, 47–50]. We divided parity into primiparity and multiparity, and reached the same conclusion after the meta-analysis [OR = 1.30, (95%CI:1.28,1.32)] (Table 1).

Five studies showed that planning pregnancy was not related to EGWG [13, 20, 40, 41, 47], and meta-analysis also showed the same result [OR = 0.92, (95%CI:0.79,1.07)] (Table 1).

The number of antenatal care procedures can affect the weight gain of pregnant women [20]. Pregnant women with adequate antenatal care were at risk for EGWG [39, 41]. However, other studies suggested that the level of antenatal care was not significantly related to EGWG [13, 24, 36, 40, 49, 51]. The findings of our meta-analysis showed that pregnant women with inadequate antenatal care had a low probability of developing EGWG (OR, 0.68; 95% CI, 0.55 − 0.84) (Table 1).

#### Illness or complications

The presence of gestational diseases or complications also increased the probability of EGWG [10, 36, 48, 51]. Pregnant women with lower limb edema were more likely to have EGWG [19]. However, He et al. found that preeclampsia was not related to EGWG [52].

### Family factors

#### Family characteristics

Pregnant women with a lower socioeconomic status were reported to more likely to have EGWG [12, 40], but Mariana et al. believed that a higher level of family economic income was a risk factor for EGWG [19]; however, 11 studies considered that level of family income was not associated with EGWG [2, 20, 22, 24, 29, 30, 41, 50, 51, 53].

#### Marital status

Some studies suggested that being unmarried or living alone was a risk factor for EGWG [10, 40, 45]; however, other studies had a different opinion [39, 49, 51]. The findings of our meta-analysis showed that pregnant women who were unmarried or lived alone were more likely to develop EGWG (OR, 1.20; 95% CI, 1.18 − 1.23) (Table 1).

#### Domestic violence

Domestic violence was considered a risk factor for EGWG [20]. However, partner abuse during pregnancy was not significantly associated with gestational weight gain [36].

#### Family support

Several studies have mentioned the impact of family support on EGWG, including food security [20, 21, 24, 32]and the housing environment [20, 24]. There was a positive correlation between food security and pregnancy weight gain in one study [20], while the other studies did not find a significant association [21, 24, 32]. The result of our meta-analysis showed that the OR of EGWG with family support was 1.13 (95% CI, 0.84 − 1.50; *P* = 0.42) (Table 1).

### Social factors

#### Race/ethnicity and culture

In America, Caucasians were more likely to develop EGWG than Blacks [39, 47, 49, 54, 55], Latinos [26, 49, 56], and Hispanics [39, 54], but some studies did not show difference between different races and EGWG [21, 36, 43]. Several studies found that race was not associated with EGWG in Canada [40, 50]and Brazil [22]. Another report indicated that Asian women had a higher risk of EGWG than Caucasian women [57], while a study of Asian populations showed that Malaysian women had a higher risk of EGWG than Chinese women [11]. A Dutch study showed a higher prevalence of EGWG in Europeans [29]. Two studies in China reported that ethnic minority status had no relationship with EGWG [2], while a study in Belgium showed that EGWG occurred more frequently in ethnic minorities [10].

#### Social support

One study reported that social support was found to be associated with weight gain during pregnancy [20], while another reported that there was no significant association [58]. Furthermore, different obstetric institutions or obstetric practitioners did not have a significant relationship with EGWG [19, 26, 50, 51]. Guidance on nutrition for EGWG was discussed in six studies [13, 21, 24, 41, 50, 51], and the results of the meta-analysis showed an OR of 1.08 (95% CI, 0.88 − 1.32) (Table 1).

#### Policy

Several studies discussed the relationship between the payment mode of medical expenses and EGWG during pregnancy [12, 23, 26, 36, 49]. Two studies found that pregnant women without medical insurance were more likely to develop EGWG [11, 23]; however, others suggested that there was no correlation [12, 26, 36].

#### Others

Persistently low neighborhood deprivation was demonstrated to reduce the risk of developing EGWG [56]. Neighborhood socioeconomic disadvantage had no statistically significant relationship with EGWG [59].

The results of some factors for EGWG are presented in Additional File 4. All forest plots of pooled ORs for various risk factors were shown in Additional File 5.

### Analysis of heterogeneity

**Fig. 3:**
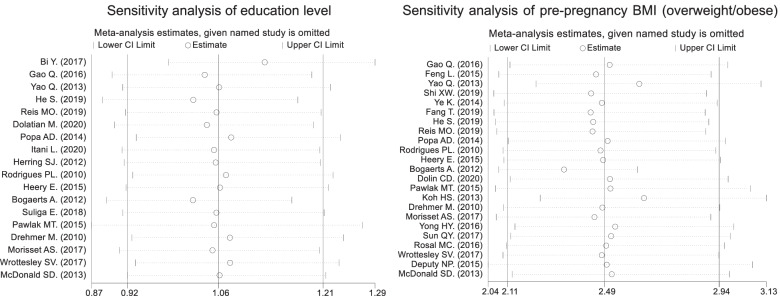
The results of sensitivity analysis

The consistency of education level and prepregnancy BMI (overweight/obesity) was confirmed by leave‐one‐out sensitivity analysis. However, the combined OR and 95% CI were not significantly affected in any of the study groups, and the difference was not statistically significant. This indicated that the above meta-analysis was stable and reliable (Fig. 3).

For educational level and prepregnancy overweight (including obesity), the above meta‐analysis was also performed within subgroups of studies, defined by the region (i.e., Africa and Asia vs. Europe and Americas), type of study (i.e., cohort study, case–control study, and cross-sectional study), and years (i.e., ≤ 2015 vs. > 2015). The subgroup analysis of the education level, combined with the meta-regression results (*P* < 0.05), suggested that heterogeneity may be related to different types of studies (Table 2).

**Table 2:** Subgroup analysis and meta-regression of factors for excessive gestational weight gain

Factors	Subgroups	Group standard	Number	Z	*P* value	I^2^	Tau^2^	OR (95%CI)	Meta-regression
Educational level	Years	≤ 2015	9	0.40	0.001*	95.7	0.0420	1.04 (0.87,1.24)	*P* = 0.693
		> 2015	9	0.55	0.001*	83.6	0.2082	1.10 (0.78,1.56)	
	Types	Cross-sectional study	5	1.14	0.017	66.7	0.1376	0.78 (0.50,1.20)	*P* = 0.020
		Cohort study	13	1.93	0.001*	93.9	0.0414	1.16 (1.00,1.34)	
	Region	Africa and Asia	7	0.32	0.001*	87.4	0.1764	1.06 (0.75,1.49)	*P* = 0.983
		Europe and America	11	0.67	0.001*	94.6	0.0427	1.06 (0.89,1.27)	
Pregnancy BMI with overweight	Years	≤ 2015	12	6.99	0.001*	98.6	0.1126	2.25 (1.79,2.94)	*P* = 0.271
		> 2015	11	10.20	0.027	50.7	0.0544	2.82 (2.31,3.44)	
	Region	Africa and Asia	11	4.15	0.001*	89.0	0.3660	2.28 (1.54,3.36)	*P* = 0.270
		Europe and America	12	9.21	0.001*	98.4	0.0985	2.78 (2.24,3.46)	
	Types	Cohort study	13	7.78	0.001*	98.4	0.1744	2.74 (0.75,3.54)	*P* = 0.640
		Case–control study	4	1.77	0.001*	83.2	0.2764	1.70 (0.95,3.04)	
		Cross-sectional study	6	6.25	0.04	57.0	0.0702	2.65 (1.95,3.59)	

* *P* < 0.001

### Analysis of publication bias

For determinants with more than 10 included studies, a funnel plot was used to evaluate publication bias. The number and distribution of each point on both sides were symmetrical in the funnel plot. Egger's test results showed that all *P* values were greater than 0.05, suggesting that there was no publication bias. The results are shown in Additional File 6.

## Discussion

The serious consequences of EGWG have been confirmed in several studies. Seventy studies with more than 3.3 million participants were included in our study. The global prevalence of EGWG is approximately 45.5%. Among the different regions, the prevalence of EGWG was highest in Americas and Africa, followed by Oceania and Europe, and the lowest was in Asia. The researches were mainly distributed in the United States and China. All of them were observational, and more than two-thirds were cohort studies. The overall quality of the included studies was relatively high. This review analyzed the determinants of EGWG in qualitative and quantitative methods using the medium to high levels of evidence. Finally, we summarized 58 determinants of EGWG from individual, family, and social themes. These results suggest that further research is required to clarify whether those variables serve as modifiable risk factors, as the identification of modifiable risk factors is essential for the development of prevention strategies.

### The determinants for EGWG

The risk factors for EGWG included the prepregnancy overweight (including obesity), younger age, unemployment, primiparity, smoking, and unmarried (including divorced), while protective factors included prepregnancy underweight and inadequate antenatal care. There was no significant correlation between EGWG and education level, alcohol consumption, planning pregnancy, food security, and access to nutrition guidance during pregnancy.

Prepregnancy BMI was the most concerning factor. Women with prepregnancy overweight (obesity) had a higher baseline weight and were more likely to experience EGWG [3], which was similar to the findings of our study. It is recommended to use an individualized approach to counsel a woman about pregnancy weight gain goals based on the woman's prepregnant BMI and track weight gain throughout the pregnancy by evaluating maternal weight at each visit [60]. For example, women with a high BMI should be encouraged to lose weight before they are ready to conceive. During preconception counseling, they should be educated about the dangers of at high BMI since a higher risk of EGWG is associated with a higher BMI. Although the results of this study showed that nutrition-related advice during pregnancy was not associated with EGWG, pregnancy weight management was influenced by factors, such as diet, exercise, self-monitoring, and health education. Health education aimed only at a single direction seems insufficient, therefore, weight management interventions for pregnant women must be comprehensive.

Age during pregnancy was also a key factor, and this study showed that younger patients had a higher risk of EGWG. This may be because older women have lower anabolism than younger women during pregnancy, or because younger women are less disciplined regarding lifestyle choices [9, 61]. Almost two-thirds of the studies reported that parity was not associated with EGWG. However, the results of the meta-analysis showed that primiparas were more likely to develop EGWG. Compared with primiparas, multipara who have fertility experience may gain more knowledge about pregnancy health care and pay more attention to nutrition and exercise during pregnancy [23]. Combining the results of age and parity, we believe that primiparas who are less than 30 years old should be the focus group. For this group, emerging mobile internet technology interventions may be more acceptable, which could guide them to adopt a healthy diet and do exercise regularly.

This study also found that being unmarried or divorced was a risk factor for EGWG. It may be that women with a lack of spouse’s company and family support, and do not pay enough attention to weight management during pregnancy. Furthermore, marital status was an important predictor of psychological symptoms and perceived stress in pregnant women [62]. Necessary psychological counseling services for unmarried or divorced pregnant women and guidance to achieve reasonable weight control is suggested during pregnancy.

This showed that unemployed pregnant women were more likely to develop EGWG. Unemployed women who live at home for a long time and do not have enough daily activities during pregnancy should be educated to modulate their lifestyle during pregnancy, and especially involve in modest exercise. Additionally, smoking was associated with an increased risk of weight gain during pregnancy. It is important in health education on healthy regarding lifestyle for pregnant women, to improve their understanding of diet and exercise during pregnancy, and guide them in developing the self-monitoring skills of diet and exercise.

According to the WHO, about 10% of pregnant women worldwide have mental illness [63]. Pregnant women are in a special physiological period and a high prevalence of psychological problems, and thus, require psychological guidance. The findings of psychosocial factors in this study were similar to those of a systematic review of psychosocial factors affecting EGWG published in 2015 [64]. Moreover, the research on cognition and self-efficacy in weight management during pregnancy is increasingly popular, although several studies have showed that the cognitive level and self-efficacy of pregnant women did not influence EGWG in this study. Higher cognition level and self-efficacy contribute to a healthy lifestyle, but the reality is harsh. In China, nearly one-third of Chinese mothers believed that weight gain during pregnancy had no effect on maternal and fetal health [65]. For many pregnant women, pregnancy weight management knowledge is exaggerated, misunderstood, or forgotten [66, 67]. Increasing the publicity of EGWG and the training in weight management skills to improve a better cognition and enhance self-efficacy is also a breakthrough point of pregnancy healthcare work in the future.

In addition, the included literature investigated the impact of domestic violence on EGWG. Domestic violence is not only a public health problem but also a serious social problem. The prevalence of domestic violence among pregnant women has been reported to range from 0.9% to 20% [68], and is even higher in some developing countries or underdeveloped regions. Domestic violence not only harms the physical and mental health of pregnant women but also increases the possibility of adverse pregnancy outcomes, seriously affecting the health of the next generation [69]. Although no direct association was observed between violence and EGWG, the fitting model showed that domestic violence increased the prevalence of depression and anxiety symptoms in pregnant women, indirectly increasing the likelihood of EGWG [20]. A cross-sectional study from China provided an in-depth analysis of the determinants of domestic violence [70]. Therefore, it is necessary to create a safe environment for pregnant women.

### Advantages and limitations

Our study had a large sample size, and the determinants of EGWG were summarized comprehensively. Both qualitative description and data combination analyses were conducted. Furthermore, a meta-analysis of single group rates was also performed for the included cohort studies to obtain the global prevalence rate of EGWG comprehensively and accurately. Overall, the study used a uniform standard from the IOM for EGWG, and all included studies had a relatively high level of evidence through serious quality assessment. However, three items scored lower in cohort studies—ascertainment of exposure, assessment of outcome, and adequacy of follow-up of cohorts. In future studies, patients’ self-reports should be avoided as much as possible for the ascertainment of exposure, and fixed archival records or structured interviews are good options. For the assessment of outcomes, it is suggested to conduct a blind independent study and avoid subjective self-reporting. It is also important to strengthen the follow-up of the research, as much as possible to complete the follow-up. To obtain more accurate results, it is necessary to carry out high-quality original studies, collect quantitative data, perform an in-depth analysis of various factors, consider the confounding factors, and improve the reliability of research results in the future.

Several limitations should also be noted: (1) Due to inconsistent research contents and grouping criteria, the data could not be combined for meta-analysis of some factors. Therefore, only a simple review was conducted, impacting the results of the analysis and the generalization of the conclusions. (2) When combining BMI, different studies used different methods of BMI classification (i.e., WHO and Asian classification). The BMI was not classified according to a specific index, but combined with those defined simply as the same category. (3) To compare and explain the effect of the determinants, the relevant data were transformed into two categories, which affected the accuracy of the results. Furthermore, the dose–response relationship of the studies was not considered. (4) The results from the forest map showed a high heterogeneity among the included studies, but the sources could not be well explained. Future research must explore the sources of heterogeneity.

## Conclusion

The high prevalence of EGWG in pregnant women remains a growing public health issue, showing the importance of preventive interventions regardless of the timing of pregnancy-related weight changes throughout life. This study was based on 70 observational studies with medium and high-level evidence, and found that EGWG was associated with 58 individual, family, and social factors. The risk factors for EGWG included the prepregnancy overweight (including obesity), younger age, unemployment, primiparity, smoking, and being unmarried (including divorced) in the meta-analysis. Protective factors that yielded some support included prepregnancy underweight and inadequate antenatal care. Therefore, it is important to avoid exposure to risk factors from multiple perspectives and develop pregnancy weight management interventions around the identified factors. Future solutions in EGWG likely require a greater focus on behavior change and maintenance.

## Supplementary Information

**Additional file 1.** Search strategy.

**Additional file 2.** Basic information of included literature.

**Additional file 3.** Quality assessment of included studies.

**Additional file 4.** Supplementary findings.

**Additional file 5.** Forest plots of pooled ORs in various factors.

**Additional file 6.** The results of publication bias.

## Data Availability

The datasets used and/or analyzed during the current study are available from the corresponding author on reasonable request.

## Abbreviations

EGWG: Excessive Gestational Weight Gain
PRISMA: Preferred Reporting Items for Systematic Reviews and Meta-analysis
IOM: Institute of Medicine
WOS: Web of Science
CNKI: China National Knowledge Internet
VIP: The VIP Database for Chinese Technical Periodicals
NOS: Newcastle − Ottawa Scale
AHRQ: Agency for Healthcare Research and Quality
OR: Odds Ratio
RR: Risk Ratio
CI: Confidence Interval
SDH: Social determinants of health
