# Supplementary material for: Determinants of excessive gestational weight gain: a systematic review and meta-analysis
Source: Arch Public Health. 2022 May 3;80:129. doi: 10.1186/s13690-022-00864-9 (PMC9066815; doi:10.1186/s13690-022-00864-9)
Supplement: Supplementary file 1 — Additional file 1. Search strategy. [file 13690_2022_864_MOESM1_ESM.docx]

**Additional file 1** Search strategy

**Chinese search term:**

1. 孕期增重、孕期增重过多
2. 孕期肥胖、孕期超重
3. 影响因素、危险因素

**English search term:**

1. gestational weight gain
2. excessive gestational weight gain
3. maternal obesity
4. maternal overweight
5. association factor / association factor/ influencing factor /determinant

**Search strategy:**

**Database1：CNKI** **2020.11.30**

| Sequence | Query | Result |
| --- | --- | --- |
| #1 | (SU=('孕期增重'+'孕期增重过多'+孕期肥胖'+‘孕期超重’) AND SU=(‘影响因素’+‘危险因素’)) AND (YE BETWEEN ('2009', '2020') ) | 377 |

**Database2：CSPD (WANFANG Data) 2020.11.30**

| Sequence | Query | Result |
| --- | --- | --- |
| #1 | ((主题:(孕期增重) or 主题:(孕期增重过多) or 主题:(孕期肥胖) or 主题:(孕期超重))and(主题:(影响因素) or 主题:(危险因素))) and Date:2009-* | 910 |

**Database3：SinoMed 2020.11.30**

| Sequence | Query | Result |
| --- | --- | --- |
| #1 | (("孕期增重"[常用字段:智能] OR "孕期增重过多"[常用字段:智能] OR "孕期肥胖"[常用字段:智能] OR "孕期超重"[常用字段:智能]) AND ("影响因素"[常用字段:智能] OR "危险因素"[常用字段:智能]) NOT ("循证文献"[文献类型] OR "病例报告"[文献类型] OR "综述"[文献类型])) AND 2009-2020[日期] | 234 |

**Database4：Vip Data 2020.11.30** 【20篇】

| Sequence | Query | Result |
| --- | --- | --- |
| #1 | (M=（孕期增重 OR 孕期增重过多 OR 孕期肥胖 OR 孕期超重）)AND （M=（影响因素 OR 危险因素））AND（years：（2009 TO 2020）） | 20 |

**Database5：PubMed 2020.11.30**

| Sequence | Query | Result |
| --- | --- | --- |
| #1 | ((((((((("Gestational Weight Gain"[Mesh]) OR (Weight Gain, Gestational[Title/Abstract])) OR (Pregnancy Weight Gain[Title/Abstract])) OR (Weight Gain, Pregnancy[Title/Abstract])) OR (Maternal Weight Gain[Title/Abstract])) OR (Weight Gain, Maternal[Title/Abstract])) OR (Postpartum Weight Retention[Title/Abstract])) OR (Weight Retention, Postpartum[Title/Abstract])) OR (weight gain during pregnancy[Title/Abstract])) OR (pregnancy weight gain[Title/Abstract]) | 3562 |
| #2 | excessive gestational weight gain[Title/Abstract] | 576 |
| #3 | (("Obesity, Maternal"[Mesh]) OR (Obesity in Pregnancy[Title/Abstract])) OR (Maternal Obesity[Title/Abstract]) | 3,046 |
| #4 | ((maternal overweight[Title/Abstract]) OR (overweight in pregnancy[Title/Abstract])) | 1,950 |
| #5 | #1 OR #2 OR #3 OR #4  (((((((((((("Gestational Weight Gain"[Mesh]) OR (Weight Gain, Gestational[Title/Abstract])) OR (Pregnancy Weight Gain[Title/Abstract])) OR (Weight Gain, Pregnancy[Title/Abstract])) OR (Maternal Weight Gain[Title/Abstract])) OR (Weight Gain, Maternal[Title/Abstract])) OR (Postpartum Weight Retention[Title/Abstract])) OR (Weight Retention, Postpartum[Title/Abstract])) OR (weight gain during pregnancy[Title/Abstract])) OR (pregnancy weight gain[Title/Abstract])) OR (excessive gestational weight gain[Title/Abstract])) OR ((("Obesity, Maternal"[Mesh]) OR (Obesity in Pregnancy[Title/Abstract])) OR (Maternal Obesity[Title/Abstract]))) OR ((maternal overweight[Title/Abstract]) OR (overweight in pregnancy[Title/Abstract])) | 7,851 |
| #6 | (factor* [Title/Abstract]) OR (determinant*[Title/Abstract]) | 3,669,143 |
| #7 | #6 AND #7  ((((((((((((("Gestational Weight Gain"[Mesh]) OR (Weight Gain, Gestational[Title/Abstract])) OR (Pregnancy Weight Gain[Title/Abstract])) OR (Weight Gain, Pregnancy[Title/Abstract])) OR (Maternal Weight Gain[Title/Abstract])) OR (Weight Gain, Maternal[Title/Abstract])) OR (Postpartum Weight Retention[Title/Abstract])) OR (Weight Retention, Postpartum[Title/Abstract])) OR (weight gain during pregnancy[Title/Abstract])) OR (pregnancy weight gain[Title/Abstract])) OR (excessive gestational weight gain[Title/Abstract])) OR ((("Obesity, Maternal"[Mesh]) OR (Obesity in Pregnancy[Title/Abstract])) OR (Maternal Obesity[Title/Abstract]))) OR ((maternal overweight[Title/Abstract]) OR (overweight in pregnancy[Title/Abstract]))) AND ((((risk factor*[Title/Abstract]) OR (association factor*[Title/Abstract])) OR (determinant*[Title/Abstract]) ) OR (influencing factor*[Title/Abstract])) | 1,581 |
| #8 | Filters: **Chinese, English, from 2009 - 2020** | 1167 |

**Database6: Web of Science 2020.11.30**

| Sequence | Query | Result |
| --- | --- | --- |
| #1 | TS=(”gestational weight gain” OR “Weight Gain, Gestational” OR “Pregnancy Weight Gain” OR “Weight Gain, Pregnancy” OR “Maternal Weight Gain” OR “Weight Gain, Maternal” OR “Postpartum Weight Retention” OR “Weight Retention, Postpartum”） | 6,848 |
| #2 | TS=(“excessive gestational weight gain”) | 694 |
| #3 | TS=("Obesity, Maternal" OR "Obesity in Pregnancy" OR "Maternal Obesity") | 5,829 |
| #4 | TS=(“maternal overweight" OR "overweight in pregnancy") | 527 |
| #5 | #1 OR #2 OR #3 OR #4 | 11,984 |
| #6 | TS=("risk factor*" OR "association factor*" OR "influencing facor*" determinant*) | 1,567,439 |
| #7 | #5 AND #6 | 3,724 |
| #8 | [排除] 文献类型: ( ABSTRACT OR EARLY ACCESS OR BOOK OR LETTER OR CORRECTION OR REVIEW OR CASE REPORT OR NEWS OR MEETING OR EDITORIAL OR REFERENCE MATERIAL ) AND 语种: ( ENGLISH OR CHINESE )数据库= SCIELO, WOS, MEDLINE, KJD, CSCD, RSCI 时间跨度=2009-2020 | 2,258 |

**Database****7: Embase 2020.11.30**

| Sequence | Query | Result |
| --- | --- | --- |
| #1 | 'gestational weight gain'/exp | 2,736 |
| #2 | 'gestational weight gain':ab,ti OR 'weight gain, gestational':ab,ti OR 'pregnancy weight gain':ab,ti OR 'weight gain, pregnancy':ab,ti OR 'maternal weight gain':ab,ti OR 'weight gain, maternal':ab,ti OR 'postpartum weight retention':ab,ti OR 'weight retention, postpartum':ab,ti OR 'weight gain during pregnancy':ab,ti | 7,322 |
| #3 | 'excessive gestational weight gain':ab,ti | 787 |
| #4 | 'maternal obesity'/exp | 5,097 |
| #5 | 'maternal obesity':ab,ti OR 'obesity in pregnancy':ab,ti OR 'obesity, maternal':ab,ti | 4,756 |
| #6 | 'maternal overweight':ab,ti OR 'overweight in pregnancy':ab,ti | 545 |
| #7 | #1 OR #2 OR #3 OR #4 OR #5 OR #6 | 14,135 |
| #8 | 'risk factor'/exp | 1,067,437 |
| #9 | 'risk factor*':ab,ti OR 'association factor*':ab,ti OR 'influencing factor*':ab,ti OR determinant*:ab,ti | 1,175,534 |
| #10 | #8 OR #9 | 1,664,573 |
| #11 | #7 AND #10 | 3,783 |
| #12 | #11 AND (2009:py OR 2010:py OR 2011:py OR 2012:py OR 2013:py OR 2014:py OR 2015:py OR 2016:py OR 2017:py OR 2018:py OR 2019:py OR 2020:py OR 2021:py) AND ('case control study'/de OR 'cohort analysis'/de OR 'cross sectional study'/de OR 'human'/de OR 'observational study'/de OR 'prospective study'/de OR 'retrospective study'/de) AND 'article'/it | 1,920 |

**Database****8: Cochrane Trail library 2020.11.30**

| Sequence | Query | Result |
| --- | --- | --- |
| #1 | Mesh descriptor: [Gestational Weight Gain] explode all trees | 54 |
| #2 | (Weight Gain, Gestational):ti,ab,kw OR (Pregnancy Weight Gain):ti,ab,kw OR (Weight Gain, Pregnancy):ti,ab,kw OR (Maternal Weight Gain):ti,ab,kw OR (Weight Gain, Maternal):ti,ab,kw (Word variations have been searched) | 2,546 |
| #3 | (excessive gestational weight gain):ti,ab,kw (Word variations have been searched) | 340 |
| #4 | Mesh descriptor: [ Obesity, Maternal] explode all trees | 10 |
| #5 | (Obesity in Pregnancy):ti,ab,kw OR (Maternal Obesity):ti,ab,kw (Word variations have been searched) | 2,443 |
| #6 | (Maternal Overweight):ti,ab,kw OR (Overweight in Pregnancy):ti,ab,kw (Word variations have been searched) | 1,025 |
| #7 | #1 OR #2 OR #3 OR #4 OR #5 OR #6 | 4,407 |
| #8 | (risk factor*):ti,ab,kw OR (association factor*):ti,ab,kw OR (influencing factor*):ti,ab,kw OR (determinant*):ti,ab,kw (Word variations have been searched) | 148,500 |
| #9 | #7 AND #8 | 941 |
| #10 | Years：2009 To 2020 | 825 |
