# Supplementary material for: Determinants of excessive gestational weight gain: a systematic review and meta-analysis
Source: Arch Public Health. 2022 May 3;80:129. doi: 10.1186/s13690-022-00864-9 (PMC9066815; doi:10.1186/s13690-022-00864-9)
Supplement: Supplementary file 2 — Additional file 2. Basic information of included literature. [file 13690_2022_864_MOESM2_ESM.docx]

**Additional file 2** Basic information of included literature

**a. Factors**

***A: Individual factors (A1-A37)***

**1. Demographic factors** including A1(Age), A2(Education level), A3(Employment).

**2.Physiological factors** including A4(Pre-pregnancy BMI), A5(Height), A6(Middle-upper arm circumference), A7(Menarche age), A8(Gene).

**3.Healthy lifestyle** including A9(Dietary), A10(Exercise), A11(Smoking), A12(Alcohol), A13(Sleeping time), A14(Electronic screen time).

**4.Psychological feature** including A15(Depression), A16(Anxiety), A17(Pressure), A18(Embarrassed), A19(Prefer a slim figure), A20(Psychological acculturation), A21(Psychological status).

**5.Cognition and self-efficacy** including A22(Cognition), A23(Self-efficacy).

**6. Maternal characteristics** including A24(Parity), A25(Prenatal care number), A26(The number of pregnancies), A27(Delivery way), A28(Planned pregnancy), A29(Gestational weeks), A30(Pregnancy interval), A31(The number of induced labor), A32(Persistent vomiting), A33(Exclusive breastfeeding plan), A34(Fertility treatment).

**7. Illness or complication** including A35(Gestational complication), A36(HIV), A37(Mental disease).

***B：Family factors (B1-B8)***

**1. Family characteristics** including B1(Family income), B2(Spouse's educational level and career), B3(Family population).

**2.Marital status** including B4(Marital status).

**3.Domestic violence** including B5(Domestic violence), B6(Partner abuse).

**4.Family support** including B7(Food security), B8(Housing environment).

***C：Social factors (C1-C13)***

**1. Racial/ethnic and culture** including C1(Racial/ethnic), C2(Discrimination), C3(Community violence), C4(Neighborhood pressure), C5(Migration), C6(Language preference), C7(Rural/Urban).

**2. Social support** including C8(Social support), C9(Antenatal care providers or providers), C10(Whether gain nutritional guidance).

**3. Policy** including C11(Medical insurance policy).

**4.Others** including C12(Continued community deprivation), C13(Community economic disadvantages).

**b.** In the data analysis method, 1 represents qualitative description and 2 represents quantitative analysis.

**c.** In the study type, 1 represents cohort study, 2 represents case-control study and 3 represents cross-sectional study.

**Table S1** Information of included literature

| **Study ID** | **Authors** | **Reference** | **Years** | **Samples**  **（n）** | **EGWG（n）** | **Country** | **Region** | **Study Type** | [**Statistic**](javascript:;) [**Analysis**](javascript:;) | **Quality** | **Influencing Factors** |
| --- | --- | --- | --- | --- | --- | --- | --- | --- | --- | --- | --- |
| 1 | Bi Y | ^1^ | 2017 | 1364 | 634 | China | Asia | 3 | 1,2 | 5 | A2, A9, A10, A24, B1, C1, |
| 2 | Gao Q | Unpublished data | 2016 | 828 | 397 | China | Asia | 1 | 1,2 | 8 | A1, A2, A4, A9, A10, A11, A12, A24, A26, B1, C1, |
| 3 | Feng L | Unpublished data | 2015 | 525 | 240 | China | Asia | 1 | 1,2 | 7 | A1, A4, A10, A11, A24, |
| 4 | Yin J | ^2^ | 2018 | 503 | 153 | China | Asia | 1 | 1 | 7 | A9, |
| 5 | Yao Q | ^3^ | 2013 | 1852 | 911 | China | Asia | 1 | 1,2 | 7 | A1, A2, A4, A24, |
| 6 | Yong HY | ^4^ | 2019 | 480 | 112 | Malaysian | Asia | 1 | 1 | 6 | A9, |
| 7 | Nunnery D | ^5^ | 2018 | 160 | 102 | USA | America | 1 | 1,2 | 6 | A4, A24, A28, C1, |
| 8 | Shi XW | ^6^ | 2019 | 1127 | 534 | China | Asia | 1 | 1,2 | 8 | A4, |
| 9 | Ye K | ^7^ | 2014 | 895 | 490 | China | Asia | 2 | 1,2 | 6 | A4, A9, A24, |
| 10 | Dai ZY | ^8^ | 2014 | 3391 | 1136 | China | Asia | 3 | 1,2 | 4 | A1, |
| 11 | Fang T | Unpublished data | 2019 | 530 | 228 | China | Asia | 3 | 1,2 | 7 | A4, |
| 12 | Restall A | ^9^ | 2014 | 1950 | 1443 | New Zealand | Oceania | 1 | 1 | 6 | A1, A4, A9, A10, A11, A13, A34, C5, |
| 13 | Gaillard R | ^10^ | 2013 | 6959 | 1474 | Netherlands | Europe | 1 | 1 | 7 | A1, A2, A4, A8, A9, A10, A11, A12, A24, B1, C1, |
| 14 | He S | ^11^ | 2019 | 724 | 190 | Singapore | Asia | 1 | 1,2 | 7 | A2, A4, A11, A35, |
| 15 | Reis MO | ^12^ | 2019 | 98 | 39 | Brazil | America | 3 | 1,2 | 5 | A1, A2, A3, A4, A24, A27, A35, B1, B4, C9, |
| 16 | Dolatian M | ^13^ | 2020 | 734 | 153 | Iran | Asia | 1 | 1,2 | 5 | A2, A3, A15, A16, A17, A25, A28, B1, B2, B3, B5, B7, B8, C8, |
| 17 | Popa AD | ^14^ | 2014 | 400 | 134 | Romania | Europe | 3 | 1,2 | 5 | A1, A2, A4, A9, A25, A28, B4, C7, C10, |
| 18 | Galin J | ^15^ | 2017 | 2364793 | 1177214 | USA | America | 1 | 1 | 9 | A4, C3, |
| 19 | Tovar A | ^16^ | 2012 | 952 | 440 | USA | America | 1 | 1 | 8 | A20, C5,C6, |
| 20 | Itani L | ^17^ | 2020 | 242 | 95 | United Arab Emirates | Asia | 1 | 1,2 | 7 | A1, A2, A4, A9, B1, |
| 21 | Herring SJ | ^18^ | 2012 | 94 | 39 | USA | America | 1 | 1,2 | 5 | A1, A2, A3, A4, A9, A10, A11, A13, A14, A24, B7, C1, C10, |
| 22 | Rugina C | ^19^ | 2020 | 115 | 66 | Romania | Europe | 1 | 1 | 8 | A9, |
| 23 | Reid AE | ^20^ | 2016 | 413 | 224 | USA | America | 1 | 1 | 7 | A1, A4, A15, A24, A29, C1, C2, C5, |
| 24 | Hartley E | ^21^ | 2015 | 256 | 106 | Australia | Oceania | 1 | 1 | 4 | A15, A16, A17, A22, |
| 25 | Rodrigues PL | ^22^ | 2010 | 173 | 38 | Brazil | America | 1 | 1,2 | 8 | A1, A2, A3, A4, A5, A7, A9, A10, A11, A12, A24, A29, A30, B1, B4, C1, |
| 26 | Heery E | ^23^ | 2015 | 799 | 499 | Ireland | Europe | 1 | 1,2 | 5 | A1, A2, A3, A4, A5, A9, A10, A11, A12, A13, A15, A17, A24, B4, C11, |
| 27 | Bogaerts A | ^24^ | 2012 | 54022 | 18619 | Belgium | Europe | 1 | 1,2 | 8 | A1, A2, A3, A4, A24, A29, A31, A35, B4, C7, |
| 28 | Vehmeijer FL | ^25^ | 2020 | 3393 | 1310 | Netherlands | Europe | 1 | 1 | 6 | A15, A16, |
| 29 | Dolin CD | ^26^ | 2020 | 508 | 143 | USA | America | 2 | 1,2 | 6 | A1, A3, A4, A9, A10, A14, A15, A24, A25, B1, B4, B7, B8, C4, C5, C10, |
| 30 | Guo YF | ^27^ | 2019 | 74424 | 42574 | Canada | America | 1 | 1 | 7 | A4, C1, |
| 31 | Ng CM | ^28^ | 2019 | 444 | 95 | Malaysia | Asia | 3 | 1 | 7 | A4, A6 |
| 32 | Suliga E | ^29^ | 2018 | 458 | 151 | Poland | Europe | 3 | 1,2 | 5 | A1, A2, A4, A9, A24, A32, B7, C7, |
| 33 | Headen I | ^30^ | 2018 | 5690 | 2448 | USA | America | 1 | 1 | 7 | C1, C12 |
| 34 | Power ML | ^31^ | 2018 | 18217 | 9637 | USA | America | 1 | 1 | 7 | A1, A4, A24, A29, A35, |
| 35 | Kominiarek MA | ^32^ | 2018 | 725 | 315 | USA | America | 1 | 1 | 7 | A10, |
| 36 | Ledoux T | ^33^ | 2018 | 159 | 81 | USA | America | 1 | 1 | 7 | A22, |
| 37 | Jersey SJ | ^34^ | 2017 | 715 | 226 | Australia | Oceania | 1 | 1 | 6 | A22, C8, |
| 38 | Gay CL | ^35^ | 2017 | 128 | 76 | USA | America | 3 | 1 | 9 | A4, A13, |
| 39 | Holowko N | ^36^ | 2015 | 163352 | 53657 | Sweden | Europe | 1 | 1 | 8 | A4, |
| 40 | Pawlak MT | ^37^ | 2015 | 230698 | 102835 | USA | America | 1 | 1,2 | 8 | A1, A2, A4, A11, A12, A24, A25, B4, C1, |
| 41 | Mendez DD | ^38^ | 2014 | 55608 | 30584 | USA | America | 1 | 1 | 7 | C13 |
| 42 | Fraga SA | ^39^ | 2014 | 1079 | 528 | Brazil | America | 3 | 1,2 | 7 | A1, A2, A4, A11, A24, A25, A35, B1, B4, C9, C10, |
| 43 | Lisa CT | ^40^ | 2014 | 1276 | 662 | USA | America | 1 | 1 | 8 | A3, A10, |
| 44 | Sangi-Haghpeykar H | ^41^ | 2014 | 282 | 126 | USA | America | 3 | 1 | 5 | A4, A15, A17, A18, A22, B4, C5, |
| 45 | Rebecca AK | ^42^ | 2013 | 4619 | 2993 | USA | America | 1 | 1 | 5 | A4, A24, A25, B4, C1, |
| 46 | Koh H | ^43^ | 2013 | 1166 | 422 | Asia | Asia | 2 | 1,2 | 6 | A1, A4, A5, A10, A11, A24, C1, C11, |
| 47 | Kowal C | ^44^ | 2012 | 74523 | 36293 | Canada | America | 2 | 1,2 | 5 | A1, A2, A4, A11, A12, A24, A25, A26, A28, A33, B1, B4, C1, C5, |
| 48 | Mehta UJ | ^45^ | 2011 | 1192 | 1015 | USA | America | 1 | 1 | 5 | A19, |
| 49 | Drehmer M | ^46^ | 2010 | 667 | 299 | Brazil | America | 1 | 1,2 | 8 | A1, A2, A4, A9, A11, A12, A24, A25, A28, B1, C10, |
| 50 | Morisset AS | ^47^ | 2017 | 913 | 586 | Canada | America | 1 | 1,2 | 6 | A1, A2, A4, A24, B1, C5, |
| 51 | Yong HY | ^48^ | 2016 | 304 | 64 | Malaysia | Asia | 3 | 1,2 | 4 | A4, A5, A10, |
| 52 | Cohen AK | ^49^ | 2016 | 6344 | 2825 | USA | America | 1 | 1 | 5 | A2, |
| 53 | Ebrahimi F | ^50^ | 2015 | 308 | 108 | Iran | Asia | 3 | 1 | 4 | A1, |
| 54 | NoorFarhana MF | ^51^ | 2015 | 422 | 55 | Malaysia | Asia | 2 | 1 | 7 | A4, |
| 55 | Shin D | ^52^ | 2014 | 490 | 258 | USA | America | 3 | 1 | 8 | A9, |
| 56 | Holowko N | ^53^ | 2014 | 4080 | 1877 | Sweden | Europe | 1 | 1 | 7 | A2, |
| 57 | Abeysena C | ^54^ | 2010 | 578 | 52 | Sri Lanka | Asia | 1 | 1 | 7 | A1, A2, A5, A10, A11, A17, A21, |
| 58 | Sun QY | ^55^ | 2017 | 1563 | 1007 | China | Asia | 2 | 1,2 | 6 | A4, |
| 59 | Rosal MC | ^56^ | 2016 | 571 | 329 | USA | America | 1 | 1,2 | 8 | A1, A3, A4, A11, A12, A24, A37, B4, C1, C9, C11, |
| 60 | Lai JS | ^57^ | 2019 | 960 | 615 | Singapore | Asia | 1 | 1 | 6 | A9, |
| 61 | Wrottesley SV | ^58^ | 2017 | 538 | 297 | South Africa | Africa | 1 | 1,2 | 5 | A1, A2, A4, A9, A11, A24, A36, B4, |
| 62 | Liu JH | ^59^ | 2014 | 133849 | 38540 | Columbia | America | 2 | 1 | 7 | C1, |
| 63 | Molyneaux E | ^60^ | 2016 | 13314 | 7956 | UK | Europe | 1 | 1 | 6 | A15, |
| 64 | Deputy NP | ^61^ | 2015 | 44421 | 20046 | USA | America | 3 | 1,2 | 7 | A1, A2, A4, A10, A12, A15, A17, A24, A25, A32, A35, B4, B6, C1, C10, C11, |
| 65 | Paulino DS | ^62^ | 2014 | 290 | 91 | Brazil | America | 1 | 1 | 7 | A24, |
| 66 | McDonald SD | ^63^ | 2013 | 330 | 190 | USA | America | 3 | 1,2 | 6 | A1, A2, A4, A9, A10, A11, A13, A14, A22, A24, B1, B4, C1, C9, |
| 67 | Koleilat M | ^64^ | 2012 | 23840 | 5698 | USA | America | 1 | 1,2 | 6 | A1, A4, B1, C6, C11, |
| 68 | Jiang H | ^65^ | 2012 | 862 | 473 | China | Asia | 1 | 1 | 7 | A10, |
| 69 | Barebring L | ^66^ | 2016 | 95 | 27 | Sweden | Europe | 1 | 1 | 6 | A9, |
| 70 | Fontaine PL | ^67^ | 2012 | 2760 | 1332 | USA | America | 1 | 1 | 7 | C1, |

**References:**

1. Bi Y, Duan YF, Wang J, Jiang S, Pang XH, Yin SA, et al. Status and related factors for gestational weight gain of Chinese pregnant women during 2010-2012. Chin J Prevent Med. 2018; 52(1): 26-30(in Chinese).
2. Yin J, Chen YY, Gong YH, Zhou R, Li M, Zhong HN, et al. Relationship between dietary energy intake and energy density and weight gain during pregnancy in pregnant women in Chengdu. J Hyg Res. 2018; 47(06): 906-912 (In Chinese).
3. Yao Q. Analysis of pre-pregnancy weight and pregnancy weight gain of pregnant women in Shaoyang City. Chin Matern Child Health Care. 2013; 28(35): 5790-5791(In Chinese).
4. Yong HY, Shariff Z, Yusof BN, Rejali Z, Tee YYS, Bindels J, et al. Pre-pregnancy BMI influences the association of dietary quality and gestational weight gain: The SECOST Study. Int J Environ Res Public Health. 2019;16(19).
5. Nunnery D, Ammerman A, Dharod J. Predictors and outcomes of excess gestational weight gain among low-income pregnant women. Health Care Women Int. 2018;39(1):19-33.
6. Shi XW, Yue J, Lyu M, Wang L, Bai E, Tie LJ. Influence of pre-pregnancy parental body mass index, maternal weight gain during pregnancy, and their interaction on neonatal birth weight. Chin J Contemp Pediatr. 2019; 21(08): 783-788(In Chinese).
7. Ye K, Zhang D, Li Y, Shen Y, Du QJ, Bo QL, et al. The influence of overweight and obesity before pregnancy and diet on weight gain during pregnancy. Chin Matern Child Health Care. 2014; 29(22): 3583-3586(In Chinese).
8. Dai ZY, Li M, Rui L, Sun XH, Pang XH, Zhou L, et al. Evaluation of pre-pregnancy weight and pregnancy weight gain among urban and rural women in southwest China. J Hyg Res. 2014; 43(04): 546-549(In Chinese).
9. Restall A, Taylor RS, Thompson JM, Flower D, Dekker GA, Kenny LC, et al. Risk factors for excessive gestational weight gain in a healthy, nulliparous cohort. J Obes. 2014;2014:148391.
10. Gaillard R, Durmus B, Hofman A, Mackenbach JP, Steegers EA, Jaddoe VW. Risk factors and outcomes of maternal obesity and excessive weight gain during pregnancy. Obesity (Silver Spring). 2013;21(5):1046-55.
11. He S, Allen JC, Razali NS, Win NM, Zhang JJ, Ng MJ, et al. Are women in Singapore gaining weight appropriately during pregnancy: a prospective cohort study. BMC Pregnancy Childbirth. 2019;19(1):290.
12. Reis MO, Maia de Sousa T, Oliveira MNS, Maioli TU, Dos Santos LC. Factors associated with excessive gestational weight gain among Brazilian mothers. Breastfeed Med. 2019;14(3):159-64.
13. Dolatian M, Sharifi N, Mahmoodi Z, Fathnezhad-Kazemi A, Bahrami-Vazir E, Rashidian T. Weight gain during pregnancy and its associated factors: a Path analysis. Nurs Open. 2020;7(5):1568-77.
14. Popa AD, Popescu RM, Botnariu GE. Adequate weight gain in pregnancy: an analysis of its determinants in a cross-sectional study. Srp Arh Celok Lek. 2014;142(11-12):695-702.
15. Galin J, Abrams B, Leonard SA, Matthay EC, Goin DE, Ahern J. Living in violent neighbourhoods is associated with gestational weight gain outside the recommended range. Paediatr Perinat Epidemiol. 2017;31(1):37-46.
16. Tovar A, Chasan-Taber L, Bermudez OI, Hyatt RR, Must A. Acculturation and gestational weight gain in a predominantly Puerto Rican population. BMC Pregnancy Childbirth. 2012;12:133.
17. Itani L, Radwan H, Hashim M, Hasan H, Obaid RS, Ghazal HA, et al. Dietary patterns and their associations with gestational weight gain in the United Arab Emirates: results from the MISC cohort. Nutr J. 2020;19(1):36.
18. Herring SJ, Nelson DB, Davey A, Klotz AA, Dibble LV, Oken E, et al. Determinants of excessive gestational weight gain in urban, low-income women. Womens Health Issues. 2012;22(5):e439-46.
19. Rugina C, Marginean CO, Melit LE, Giga DV, Modi V, Marginean C. Relationships between excessive gestational weight gain and energy and macronutrient intake in pregnant women. J Int Med Res. 2020;48(8):300060520933808.
20. Reid AE, Rosenthal L, Earnshaw VA, Lewis TT, Lewis JB, Stasko EC, et al. Discrimination and excessive weight gain during pregnancy among Black and Latina young women. Soc Sci Med. 2016;156:134-41.
21. Hartley E, McPhie S, Fuller-Tyszkiewicz M, Hill B, Skouteris H. Psychosocial factors and excessive gestational weight gain: the effect of parity in an Australian cohort. Midwifery. 2016;32:30-7.
22. Rodrigues PL, de Oliveira LC, Brito Ados S, Kac G. Determinant factors of insufficient and excessive gestational weight gain and maternal-child adverse outcomes. Nutrition. 2010;26(6):617-23.
23. Heery E, Kelleher CC, Wall PG, McAuliffe FM. Prediction of gestational weight gain - a biopsychosocial model. Public Health Nutr. 2015;18(8):1488-98.
24. Bogaerts A, Van den Bergh B, Nuyts E, Martens E, Witters I, Devlieger R. Socio-demographic and obstetrical correlates of pre-pregnancy body mass index and gestational weight gain. Clin Obes. 2012;2(5-6):150-9.
25. Vehmeijer FOL, Balkaran SR, Santos S, Gaillard R, Felix JF, Hillegers MHJ, et al. Psychological distress and weight gain in pregnancy: a population-based study. Int J Behav Med. 2020;27(1):30-8.
26. Dolin CD, Gross RS, Deierlein AL, Berube LT, Katzow M, Yaghoubian Y, et al. Predictors of gestational weight gain in a low-income Hispanic population: sociodemographic characteristics, health behaviors, and psychosocial stressors. Int J Environ Res Public Health. 2020;17(1).
27. Guo Y, Miao Q, Huang T, Fell DB, Harvey ALJ, Wen SW, et al. Racial/ethnic variations in gestational weight gain: a population-based study in Ontario. Can J Public Health. 2019;110(5):657-67.
28. Ng CM, Badon SE, Dhivyalosini M, Hamid JJM, Rohana AJ, Teoh AN, et al. Associations of pre-pregnancy body mass index, middle-upper arm circumference, and gestational weight gain. Sex Reprod Healthc. 2019;20:60-5.
29. Suliga E, Rokita W, Adamczyk-Gruszka O, Pazera G, Ciesla E, Gluszek S. Factors associated with gestational weight gain: a cross-sectional survey. BMC Pregnancy Childbirth. 2018;18(1):465.
30. Headen I, Mujahid M, Deardorff J, Rehkopf DH, Abrams B. Associations between cumulative neighborhood deprivation, long-term mobility trajectories, and gestational weight gain. Health Place. 2018;52:101-9.
31. Power ML, Lott ML, Mackeen AD, DiBari J, Schulkin J. A retrospective study of gestational weight gain in relation to the Institute of Medicine's recommendations by maternal body mass index in rural Pennsylvania from 2006 to 2015. BMC Pregnancy Childbirth. 2018;18(1):239.
32. Kominiarek MA, Grobman W, Adam E, Buss C, Culhane J, Entringer S, et al. Stress during pregnancy and gestational weight gain. J Perinatol. 2018;38(5):462-7.
33. Ledoux T, Daundasekara S, Van Den Berg P, Leung P, Walker L, Berens PD. Association between health beliefs and gestational weight gain. J Womens Health (Larchmt). 2018;27(3):341-7.
34. de Jersey SJ, Mallan KM, Callaway LK, Daniels LA, Nicholson JM. Prospective relationships between health cognitions and excess gestational weight gain in a cohort of healthy and overweight pregnant women. J Acad Nutr Diet. 2017;117(8):1198-209.
35. Gay CL, Richoux SE, Beebe KR, Lee KA. Sleep disruption and duration in late pregnancy is associated with excess gestational weight gain among overweight and obese women. Birth. 2017;44(2):173-80.
36. Holowko N, Chaparro MP, Nilsson K, Ivarsson A, Mishra G, Koupil I, et al. Social inequality in pre-pregnancy BMI and gestational weight gain in the first and second pregnancy among women in Sweden. J Epidemiol Community Health. 2015;69(12):1154-61.
37. Pawlak MT, Alvarez BT, Jones DM, Lezotte DC. The effect of race/ethnicity on gestational weight gain. J Immigr Minor Health. 2015;17(2):325-32.
38. Mendez DD, Doebler DA, Kim KH, Amutah NN, Fabio A, Bodnar LM. Neighborhood socioeconomic disadvantage and gestational weight gain and loss. Matern Child Health J. 2014;18(5):1095-103.
39. Fraga AC, Theme Filha MM. Factors associated with gestational weight gain in pregnant women in Rio de Janeiro, Brazil, 2008. Cad Saude Publica. 2014;30(3):633-44.
40. Chasan-Taber L, Silveira M, Lynch KE, Pekow P, Solomon CG, Markenson G. Physical activity and gestational weight gain in Hispanic women. Obesity (Silver Spring). 2014;22(3):909-18.
41. Sangi-Haghpeykar H, Lam K, Raine SP. Gestational weight gain among Hispanic women. Matern Child Health J. 2014;18(1):153-60.
42. Krukowski RA, Bursac Z, McGehee MA, West D. Exploring potential health disparities in excessive gestational weight gain. J Womens Health (Larchmt). 2013;22(6):494-500.
43. Koh H, Ee TX, Malhotra R, Allen JC, Tan TC, Ostbye T. Predictors and adverse outcomes of inadequate or excessive gestational weight gain in an Asian population. J Obstet Gynaecol Res. 2013;39(5):905-13.
44. Kowal C, Kuk J, Tamim H. Characteristics of weight gain in pregnancy among Canadian women. Matern Child Health J. 2012;16(3):668-76.
45. Mehta UJ, Siega-Riz AM, Herring AH. Effect of body image on pregnancy weight gain. Matern Child Health J. 2011;15(3):324-32.
46. Drehmer M, Camey S, Schmidt MI, Olinto MTA, Giacomello A, Buss C, et al. Socioeconomic, demographic and nutritional factors associated with maternal weight gain in general practices in southern Brazil. Cad Saude Publica. 2010; 26(5):1024–34.
47. Morisset AS, Dubois L, Colapinto CK, Luo ZC, Fraser WD. Prepregnancy Body Mass Index as a significant predictor of total gestational weight gain and birth weight. Can J Diet Pract Res. 2017;78(2):66-73.
48. Yong HY, Mohd Shariff Z, Koo SJ, Binti Sa'ari NS. Pre-pregnancy body mass index, height and physical activity are associated with rate of gestational weight gain among Malaysian mothers. J Obstet Gynaecol Res. 2016;42(9):1094-101.
49. Cohen AK, Kazi C, Headen I, Rehkopf DH, Hendrick CE, Patil D, et al. Educational attainment and gestational weight gain among U.S. mothers. Womens Health Issues. 2016;26(4):460-7.
50. Ebrahimi F, Shariff ZM, Tabatabaei SZ, Fathollahi MS, Mun CY, Nazari M. Relationship between sociodemographics, dietary intake, and physical activity with gestational weight gain among pregnant women in Rafsanjan city , Iran. J Heal Popul Nutr. 2015; 33: 168–176.
51. Farhana MFN, Rohana AJ, Alina TIT. Excessive and inadequate gestational weight gain among Malaysian pregnant women in rural area: Are There Any Associated Factors? Pakistan Journal of Nutrition. 2015;14(12):854-61.
52. Shin D, Bianchi L, Chung H, Weatherspoon L, Song WO. Is gestational weight gain associated with diet quality during pregnancy? Matern Child Health J. 2014;18(6):1433-43.
53. Holowko N, Mishra G, Koupil I. Social inequality in excessive gestational weight gain. Int J Obes (Lond). 2014;38(1):91-6.
54. Abeysena C, Jayawardana P. Maternal and social determinants of excessive weight gain during pregnancy: a cohort study. Int J Coll Res Internal Med & Public Health. 2010; 2(2): 348-359.
55. Sun QY, Cai Y. Effects of age on gestational weight gain and neonatal birth weight. Prog Obstet Gynecol. 2017; 26(03): 222-224.
56. Rosal MC, Wang ML, Moore Simas TA, Bodenlos JS, Crawford SL, Leung K, et al. Predictors of gestational weight gain among White and Latina women and associations with birth weight. J Pregnancy. 2016;2016:8984928.
57. Lai JS, Soh SE, Loy SL, Colega M, Kramer MS, Chan JKY, et al. Macronutrient composition and food groups associated with gestational weight gain: the GUSTO study. Eur J Nutr. 2019; 58(3): 1081–1094.
58. Wrottesley SV, Pisa PT, Norris SA. The influence of maternal dietary patterns on body mass index and gestational weight gain in urban Black South African women. Nutrients. 2017;9(7).
59. Liu J, Gallagher AE, Carta CM, Torres ME, Moran R, Wilcox S. Racial differences in gestational weight gain and pregnancy-related hypertension. Ann Epidemiol. 2014;24(6):441-7.
60. Molyneaux E, Poston L, Khondoker M, Howard LM. Obesity, antenatal depression, diet and gestational weight gain in a population cohort study. Arch Womens Ment Health. 2016;19(5):899-907.
61. Deputy NP, Sharma AJ, Kim SY, Hinkle SN. Prevalence and characteristics associated with gestational weight gain adequacy. Obstet Gynecol. 2015;125(4):773-81.
62. Paulino DS, Surita FG, Peres GB, do Nascimento SL, Morais SS. Association between parity, pre-pregnancy body mass index and gestational weight gain. J Matern Fetal Neonatal Med. 2016;29(6):880-4.
63. McDonald SD, Park CK, Timm V, Schmidt L, Neupane B, Beyene J. What psychological, physical, lifestyle, and knowledge factors are associated with excess or inadequate weight gain during pregnancy? A cross-sectional survey. Journal of Obstetrics and Gynaecology Canada. 2013;35(12):1071-82.
64. Koleilat M, Whaley SE. Trends and predictors of excessive gestational weight gain among hispanic WIC participants in Southern California. Matern Child Health J. 2013;17(8):1399-404.
65. Jiang H, Qian X, Li M, Lynn H, Fan YY, Jiang HY, et al. Can physical activity reduce excessive gestational weight gain? Findings from a Chinese urban pregnant women cohort study. Int J Behav Nutr Phys Act. 2012; 9(1): 12.
66. Barebring L, Brembeck P, Lof M, Brekke HK, Winkvist A, Augustin H. Food intake and gestational weight gain in Swedish women. Springerplus. 2016;5:377.
67. Fontaine PL, Hellerstedt WL, Dayman CE, Wall MM, Sherwood NE. Evaluating body mass index-specific trimester weight gain recommendations: differences between black and white women. J Midwifery Womens Health. 2012;57(4):327-35.
