# Supplementary material for: Determinants of excessive gestational weight gain: a systematic review and meta-analysis
Source: Arch Public Health. 2022 May 3;80:129. doi: 10.1186/s13690-022-00864-9 (PMC9066815; doi:10.1186/s13690-022-00864-9)
Supplement: Supplementary file 3 — Additional file 3. Quality assessment of included studies. [file 13690_2022_864_MOESM3_ESM.docx]

**Additional file 3** Quality assessment of included studies

**Table S1** Quality scores assessing the quality of included cohort studies using a Newcastle-Ottawa scale

| Author/Year | **Selection** | | | | **Comparability** | **Outcome** | | | Total |
| --- | --- | --- | --- | --- | --- | --- | --- | --- | --- |
|  | Representativeness of the exposed cohort（*） | Selection of the non-exposed cohort（*） | Ascertainment of exposure（*） | Outcome not present at the start（*） | Comparability of cohorts on basis of design or analysis（**） | Assessment of outcome（*） | Follow-up long enough for outcome（*） | Adequacy of follow-up（*） |  |
| Gao Q,2016 | 1 | 1 | 1 | 1 | 1 | 1 | 1 | 1 | 8 |
| Feng L,2015 | 1 | 1 | 1 | 1 | 1 | 1 | 1 | 0 | 7 |
| Yin J,2018 | 1 | 1 | 1 | 1 | 1 | 1 | 1 | 0 | 7 |
| Yao Q,2013 | 1 | 1 | 1 | 1 | 1 | 1 | 1 | 0 | 7 |
| Yong HY,2019 | 1 | 1 | 1 | 1 | 1 | 0 | 1 | 0 | 6 |
| Nunnery D,2018 | 1 | 1 | 1 | 1 | 1 | 0 | 1 | 0 | 6 |
| Shi XW,2019 | 1 | 1 | 1 | 1 | 1 | 1 | 1 | 1 | 8 |
| Restall A,2014 | 1 | 1 | 0 | 1 | 1 | 1 | 1 | 0 | 6 |
| Gaillard R,2013 | 1 | 1 | 0 | 1 | 1 | 1 | 1 | 1 | 7 |
| He S,2019 | 1 | 1 | 1 | 1 | 1 | 1 | 1 | 0 | 7 |
| Dolatian M,2020 | 1 | 1 | 0 | 1 | 0 | 0 | 1 | 1 | 5 |
| Galin J,2017 | 1 | 1 | 1 | 1 | 2 | 1 | 1 | 1 | 9 |
| Tovar A,2012 | 1 | 1 | 1 | 1 | 1 | 1 | 1 | 1 | 8 |
| Itani L,2020 | 1 | 1 | 1 | 1 | 1 | 0 | 1 | 1 | 7 |
| Herring SJ,2012 | 1 | 1 | 0 | 1 | 1 | 0 | 1 | 0 | 5 |
| Rugină C,2020 | 1 | 1 | 1 | 1 | 1 | 1 | 1 | 1 | 8 |
| Reid AE,2016 | 1 | 1 | 1 | 1 | 1 | 0 | 1 | 1 | 7 |
| Hartley E,2015 | 1 | 1 | 0 | 1 | 0 | 0 | 1 | 0 | 4 |
| Rodrigues PL,2010 | 1 | 1 | 1 | 1 | 1 | 1 | 1 | 1 | 8 |
| Heery E,2015 | 1 | 1 | 0 | 1 | 1 | 0 | 1 | 0 | 5 |
| Bogaerts A,2012 | 1 | 1 | 1 | 1 | 1 | 1 | 1 | 1 | 8 |
| Vehmeijer FL,2020 | 1 | 1 | 0 | 1 | 1 | 0 | 1 | 1 | 6 |
| Guo YF,2019 | 1 | 1 | 0 | 1 | 1 | 1 | 1 | 1 | 7 |
| Headen I,2018 | 1 | 1 | 1 | 1 | 1 | 0 | 1 | 1 | 7 |
| Power ML,2018 | 1 | 1 | 1 | 1 | 0 | 1 | 1 | 1 | 7 |
| Kominiarek MA,2018 | 1 | 1 | 1 | 1 | 1 | 0 | 1 | 1 | 7 |
| Ledoux T,2018 | 1 | 1 | 0 | 1 | 1 | 1 | 1 | 1 | 7 |
| Jersey SJ,2017 | 1 | 1 | 0 | 1 | 1 | 0 | 1 | 1 | 6 |
| Holowko N,2014 | 1 | 1 | 1 | 1 | 1 | 0 | 1 | 1 | 7 |
| Pawlak MT,2015 | 1 | 1 | 1 | 1 | 1 | 1 | 1 | 1 | 8 |
| Mendez DD,2014 | 1 | 1 | 1 | 1 | 1 | 0 | 1 | 1 | 7 |
| Lisa CT,2014 | 1 | 1 | 1 | 1 | 1 | 1 | 1 | 1 | 8 |
| Rebecca AK,2013 | 1 | 1 | 0 | 1 | 0 | 1 | 1 | 1 | 5 |
| Mehta UJ,2011 | 1 | 1 | 0 | 1 | 1 | 0 | 1 | 0 | 5 |
| Drehmer M,2010 | 1 | 1 | 1 | 1 | 1 | 1 | 1 | 1 | 8 |
| Morisset AS,2017 | 1 | 1 | 0 | 1 | 1 | 0 | 1 | 1 | 6 |
| Cohen AK,2016 | 1 | 1 | 0 | 1 | 1 | 0 | 1 | 0 | 5 |
| Holowko N,2014 | 1 | 1 | 1 | 1 | 1 | 1 | 1 | 1 | 8 |
| Abeysena C,2010 | 1 | 1 | 0 | 1 | 1 | 1 | 1 | 1 | 7 |
| Rosal MC,2016 | 1 | 1 | 1 | 1 | 1 | 1 | 1 | 1 | 8 |
| Lai JS,2019 | 1 | 1 | 0 | 1 | 1 | 1 | 1 | 0 | 6 |
| Wrottesley SV,2017 | 1 | 1 | 0 | 1 | 1 | 0 | 1 | 0 | 5 |
| Molyneaux E,2016 | 1 | 1 | 0 | 1 | 1 | 1 | 1 | 0 | 6 |
| Paulino DS,2014 | 1 | 1 | 1 | 1 | 1 | 1 | 1 | 0 | 7 |
| Koleilat M,2012 | 1 | 1 | 1 | 1 | 0 | 1 | 1 | 0 | 6 |
| Jiang H,2012 | 1 | 1 | 1 | 1 | 1 | 1 | 1 | 0 | 7 |
| Bärebring L,2016 | 1 | 1 | 0 | 1 | 1 | 0 | 1 | 1 | 6 |
| Fontaine PL,2012 | 1 | 1 | 0 | 1 | 1 | 1 | 1 | 1 | 7 |

**Table S2** Quality scores assessing the quality of included case-control studies using a Newcastle-Ottawa scale

| Author/Year | **Selection** | | | | **Comparability** | **Outcome** | | | Total |
| --- | --- | --- | --- | --- | --- | --- | --- | --- | --- |
|  | Adequate case definition（*） | Representativeness of the cases（*） | Selection of Controls（*） | Definition of Controls（*） | Comparability of cases and controls on basis of design or analysis（**） | Ascertainment of exposure（*） | Same method of ascertainment for cases and controls（*） | Non-Response rate（*） |  |
| Ye K,2014 | 1 | 1 | 1 | 1 | 1 | 0 | 1 | 0 | 6 |
| Dolin CD,2020 | 1 | 1 | 1 | 1 | 1 | 0 | 1 | 0 | 6 |
| Koh H,2013 | 1 | 1 | 1 | 1 | 1 | 0 | 1 | 0 | 6 |
| Kowal C,2012 | 1 | 1 | 1 | 1 | 0 | 0 | 1 | 0 | 5 |
| NoorFarhana MF,2015 | 1 | 1 | 1 | 1 | 1 | 1 | 1 | 0 | 7 |
| Sun QY,2017 | 1 | 1 | 1 | 1 | 0 | 1 | 1 | 0 | 6 |
| Liu JH,2014 | 1 | 1 | 1 | 1 | 1 | 1 | 1 | 0 | 7 |

**Table S3** Quality scores assessing the quality of included cross-sectional studies using an AHRQ scale

| **Author/Year** | **a** | **b** | **c** | **d** | **e** | **f** | **g** | **h** | **i** | **j** | **k** | **Total** |
| --- | --- | --- | --- | --- | --- | --- | --- | --- | --- | --- | --- | --- |
| Bi Y,2017 | 1 | ? | 1 | ? | 1 | 1 | ? | 1 | 0 | ? | ? | 5 |
| Dai ZY,2014 | 1 | 1 | 1 | ? | 1 | 0 | ? | 0 | ? | ? | ? | 4 |
| Fang T,2019 | 1 | 1 | 1 | 0 | 1 | 1 | 1 | 0 | ? | 1 | ? | 7 |
| Reis MO,2019 | 1 | 1 | 1 | 1 | 1 | 0 | ? | 0 | 0 | ? | ? | 5 |
| Popa AD,2014 | 1 | 1 | 1 | 1 | 1 | 0 | ? | 0 | ? | ? | ? | 5 |
| Ng CM,2019 | 1 | 1 | 1 | ? | 1 | 0 | 1 | 1 | 1 | ? | ? | 7 |
| Suliga E,2018 | 1 | 1 | 1 | 0 | 1 | 0 | 0 | 1 | 0 | ? | ? | 5 |
| Gay CL,2017 | 1 | 1 | 1 | 1 | 1 | 0 | 1 | 1 | 1 | 1 | ? | 9 |
| Fraga SA,2014 | 1 | 1 | 1 | 1 | 1 | 0 | 1 | 0 | 1 | ? | ? | 7 |
| Sangi-Haghpeykar H,2014 | 1 | 0 | 1 | 1 | 1 | 0 | ? | 1 | ? | 0 | ? | 5 |
| Yong HY,2016 | 1 | 1 | 0 | ? | 1 | 0 | ? | 1 | ? | ? | ? | 4 |
| Ebrahimi F,2015 | 1 | 1 | 0 | ? | 1 | 0 | ? | 1 | ? | ? | ? | 4 |
| Shin D,2014 | 1 | 1 | 1 | 1 | 1 | 0 | 1 | 1 | 1 | ? | ? | 8 |
| Deputy NP,2015 | 1 | 1 | 1 | 1 | 1 | 0 | ? | 1 | 1 | 0 | ? | 7 |
| McDonald SD,2013 | 1 | 1 | 1 | 0 | 1 | 0 | ? | 1 | 0 | 1 | ? | 6 |
| **a.** Define the source of information（survey or record review）. **b.** List inclusion and exclusion criteria for exposed and unexposed subjects (cases and controls) or refer to previous publications. **c.** Indicate time period used for identifying patients. **d.** Indicate whether or not subjects were consecutive if not population-based. **e.** Indicate if evaluators of subjective components of study were masked to other aspects of the status of the participants. **f.** Describe any assessments undertaken for quality assurance purposes (e.g., test/retest of primary outcome measurements). **g.** Explain any patient exclusions from analysis. **h.** Describe how confounding was assessed and/or controlled. **i.** If applicable, explain how missing data were handled in the analysis. **j.** Summarize patient response rates and completeness of data collection. **k.** Clarify what follow-up, if any, was expected and the percentage of patients for which incomplete data or follow-up was obtained. | | | | | | | | | | | | |
