# Supplementary material for: Determinants of excessive gestational weight gain: a systematic review and meta-analysis
Source: Arch Public Health. 2022 May 3;80:129. doi: 10.1186/s13690-022-00864-9 (PMC9066815; doi:10.1186/s13690-022-00864-9)
Supplement: Supplementary file 4 — Additional file 4. Supplementary findings. [file 13690_2022_864_MOESM4_ESM.docx]

**Additional file 4** Supplementary findings

**Table S1** Supplementary findings of influencing factors for EGWG

| **Factors** | **Results** | **Main findings** | **Study ID** |
| --- | --- | --- | --- |
| ***A: Individual factors A1-A37*** | | | |
| **1 Demographic factors** |  |  |  |
| A1 Age | S | Meta-analysis showed younger (≤30) was a risk factor for EGWG. | **(N=30)**2,3,5,10,12,13,15,17,20,21,23,25,26,27,29,32,34,40,42,46,47,49,50,53,57,59,61,64,66,67 |
| A2 Education level | NS | Meta-analysis exported there were no significant correlation between EGWG and education level. | **(N=25)**1,2,5,13,14,15,16,17,20,21,25,26,27,32,40,42,47,49,50,52,56,57,61,64,66 |
| A3 Employment | S | Meta-analysis found unemployed pregnant women were more likely to develop EGWG. | **(N=9)**15,16,21,25,26,27,29,43,59 |
| **2 Physiological and anthropometric factors** |  |  |  |
| A4 Pre-pregnancy BMI | S | Meta-analysis manifested women with pre-pregnancy overweight were high likely to develop EGWG as normal weight and women with pre-pregnancy underweight were about half probability to develop EGWG. | **(N=42)**2,3,5,7,8,9,11,12,13,14,15,17,18,20,21,23,25,26,27,29,30,31,32,34,38,39,40,42,44,45,46,47,49,50,51,54,58,59,61,64,66,67 |
| A5 Height | C | Two studies mentioned a higher incidence of EGWG in taller women, (26, 46) but others found no correlation. (25,51,57) | **(N=5)**25,26,46,51,57 |
| A6 Middle-upper arm circumference | S | A high middle-upper arm circumference (MUAC) was associated with a high risk for EGWG. (31) | **(N=1)**31 |
| A7 Menarche age | S | Pregnant women who had menarche before 12 years old were more likely to experience EGWG. (25) | **(N=1)**25 |
| A8 Gene | NS | A study examined that the gene of FTO rs8050136 was not associated with EGWG. (13) | **(N=1)**13 |
| **3 Healthy lifestyle** |  |  |  |
| A9 Dietary | C | This was a controversial factor, the specific content can be seen in the paper. Some studies were not observed the influence of dietary factors on EGWG (1,35,9,25,35,49,29,66).The supplementation of Folic acid (13) and DHA (46) during pregnancy was not related to EGWG, either. | **(N=21)**1,2,4,6,9,12,13,17,20,21,22,25,26,29,32,49,55,60,61,66,69 |
| A10 Exercise | C | This was a controversial factor, the specific content can be seen in the paper. Several studies showed no association between pregnancy exercises and EGWG (2,25,26,35,43,57,66). Two studies showed that sedentary behavior during pregnancy was not associated with EGWG, either (2,43). Particularly, women exposed to passive smoking in the third trimester can reduce the risk for EGWG (57). | **(N=17)**1,2,3,12,13,21,25,26,29,35,43,46,51,57,64,66,68 |
| A11 Smoking | S | Smokers (3,13,14,25,40) and ex-smokers (12,32,64) had a higher risk for EGWG than those who had never smoked.Ten studies demonstrated no association between EGWG and smoking status (2,21,26,42,46,47,49,59,61,66). The probability of EGWG in pregnant women with smoking was 1.29 times higher than that without smoking. | **(N=17)**2,3,12,13,14,21,25,26,40,42,46,47,49,57,59,61,66 |
| A12 Alcohol | NS | Meta-analysis exported there were no significant correlation between EGWG and alcohol. | **(N=9)**2,13,25,26,40,47,49,59,64 |
| A13 Sleeping time | C | Five studies (12,21,26,38,66) inspected the relationship between sleep duration and EGWG. Three of them (21,26,66) believed that sufficient sleep duration had nothing to do with EGWG. However, the study (12) displayed that a sleep duration of more than 8 hours at night was more likely to cause EGWG, and longer sleep duration during the day was also a risk factor. (38) | **(N=5)**12,21,26,38,66, |
| A14 Electronic screen time | S | Electronic screen time during pregnancy is also important. Low TV viewing during pregnancy was a protective factor for EGWG, (21) while bedtime TV viewing can increase the risk of EGWG. (66) | **(N=3)**21,29,66, |
| **4 Psychological feature** |  |  |  |
| A15 Depression | C | This was a controversial factor, the specific content can be seen in the paper. | **(N=9)**16,23,24,26,28,29,44,63,64 |
| A16 Anxiety | C | This was a controversial factor, the specific content can be seen in the paper. | **(N=3)**16,24,28, |
| A17 Pressure | C | This was a controversial factor, the specific content can be seen in the paper. | **(N=6)**16,24,26,44,57,64, |
| A18 Embarrassed | S | A study demonstrated that pregnant women who were embarrassed when weighing themselves were at higher risk of developing EGWG. (44) | **(N=1)**44 |
| A19 Prefer a slim figure | S | A study demonstrated that pregnant women who preferred to be thinner (48) were at higher risk of developing EGWG. | **(N=1)**48 |
| A20 Psychological acculturation | NS | This factor was not associated with EGWG. | **(N=1)**19 |
| A21 Psychological status | NS | This factor was not associated with EGWG. | **(N=1)**57 |
| **5** **Cognition and self-efficacy** |  |  |  |
| A22 Cognition | C | Four studies (24,37,44,66) about cognitive levels of a healthy lifestyle, referring to food intake control, nutrition, physical exercise, vitamin supplementation, and breastfeeding displayed no effect on EGWG. | **(N=5)**24,36,37,44,66 |
| A23 Self-efficacy | S | Pregnant women with low self-efficacy of weight management was a risk factor for EGWG. (24) | **(N=1)**24 |
| **6** **Maternal characteristics** |  |  |  |
| A24 Parity | S | Meta-analysis of 18 studies came to primipara are more likely to develop EGWG. | **(N=28)**1,2,3,5,7,9,13,15,21,23,25,26,27,29,32,34,40,42,45,46,47,49,50,59,61,64,65,66 |
| A25 Prenatal care number | S | Meta-analysis showed pregnant women with adequate antenatal care had a high likelihood to develop EGWG. | **(N=9)**16,17,29,40,42,45,47,49,64, |
| A26 The number of pregnancies | NS | Other studies referred to the number of pregnancies was not related to EGWG. (2,47) | **(N=2)**2,47, |
| A27 Delivery way | S | Pregnant women who had cesarean section were more likely to develop EGWG. (15) | **(N=3)**15, |
| A28 Planned pregnancy | NS | Meta-analysis exported there were no significant correlation between EGWG and planning pregnancy. | **(N=5)**7,16,17,47,49, |
| A29 Gestational weeks | S | Longer gestational age may be associated with higher EGWG. (23,25,27,34) | **(N=4)**23,25,27,34, |
| A30 Pregnancy interval | NS | The interval of pregnancy was not related to EGWG. (25) | **(N=1)**25 |
| A31 The number of induced labor | NS | The number of induced labor was not related to EGWG. (27) | **(N=1)**27 |
| A32 Persistent vomiting | C | Persistent vomiting during pregnancy was a protective factor for the occurrence of EGWG in pre-pregnancy overweight women, (64) but Edyta found no correlation. (32) | **(N=2)**32,64 |
| A33 Exclusive breastfeeding plan | NS | The plan of exclusive breastfeeding was not related to EGWG. (47) | **(N=1)**47 |
| A34 Fertility treatment | S | Receiving fertility treatment was a protective factor for EGWG. (12) | **(N=1)**12 |
| **7** **Illness or complication** |  |  |  |
| A35 Gestational complication | C | The presence of gestational diseases or complications increased the likelihood of EGWG (27,34,42,64). Other studies found no correlation between complications and EGWG. | **(N=6)**14,15,27,34,42,64 |
| A36 HIV | S | Pregnant women with HIV were regarded as a protective factor to reducing the risk of developing EGWG. (61) | **(N=1)**61 |
| A37 Mental disease | NS | In addition, pregnant women with mental illness made no impact on EGWG. (59) | **(N=1)**59 |
| ***B：Family factors B1-B8*** | | | |
| **1 Family characteristics** |  |  |  |
| B1 Family income | C | Most studies considered that without connection. (1,2,13,16,20,25,29,42,49,50,66) | **(N=14)**1,2,13,15,16,20,25,29,42,47,49,50,66,67 |
| B2 Spouse's educational level and career | NS | This factor was not associated with EGWG. | **(N=1)**16 |
| B3 Family population | S | The family size was positively correlated with EGWG. (16) | **(N=1)**16 |
| **2** **Marital status** |  |  |  |
| B4 Marital status | S | Meta-analysis showed that pregnant women who were unmarried or living alone were more likely to develop EGWG. | **(N=15)**15,17,25,26,27,29,40,42,44,45,47,59,61,64,66 |
| **3 Domestic violence** |  |  |  |
| B5 Domestic violence | S | Domestic violence was associated with an increased risk of EGWG. (16) | **(N=1)**16 |
| B6 Partner abuse | NS | partner abuse during pregnancy was not associated with gestational weight gain. (64) | **(N=1)**64 |
| **4 Family support** |  |  |  |
| B7 Food security | NS | Meta-analysis exported there were no significant correlation between EGWG and food security. | **(N=4)**16,21,29,32 |
| B8 Housing environment | NS | This factor was not associated with EGWG. | **(N=2)**16,29 |
| ***C：Social factors C1-C13*** | | | |
| **1** **Racial/ethnic Culture** |  |  |  |
| C1 Racial/ethnic | C | This was a controversial factor, the specific content can be seen in the paper. | **(N=18)**1,2,7,13,21,23,25,30,33,40,45,46,47,59,62,64,66,70 |
| C2 Discrimination | S | Women who had experienced discrimination were 71 percent more likely to be overweight. (23) | **(N=1)**23 |
| C3 Community violence | S | EGWG was associated with higher levels of community violence. (18) | **(N=1)**18 |
| C4 Neighborhood pressure | NS | A study reported that neighborhood pressure was not associated with EGWG. (29) | **(N=1)**29 |
| C5 Migration | C | For the migration, the EGWG was not affected by emigrating (47), the length of residence in the emigrated place, (29) whether born in the emigrated place, (23,29,44,50) or the language habits. (19,29) However, a higher rate of EGWG occurred in those who have immigrated to the United States for a shorter time (12) and a higher rate of EGWG in Latino women or their family members born in the United States. (19) | **(N=7)**12,19,23,29,44,47,50 |
| C6 Language preference | C | Spoken language preference were not significantly associated with EGWG (19). A lower rate of EGWG in Hispanic women than in English speakers (67). | **(N=1)**19,67 |
| C7 Rural/Urban | C | Two studies showed no difference in urban and rural areas, (17,32) but one study indicated a higher incidence of EGWG in the urban area. (27) | **(N=3)**17,27,32 |
| **2** **Social support** |  |  |  |
| C8 Social support | C | One study observed an association between social support and EGWG, (16) while the other one found no significance. (37) Additionally, pregnant women were more likely to develop EGWG if they received advice about weight gain that was not based on IOM guidelines. (21) | **(N=2)**16,37 |
| C9 Antenatal care providers or providers | NS | This factor was not associated with EGWG. | **(N=4)**15,42,59,66 |
| C10 Whether gain nutritional guidance | NS | Meta-analysis exported there were no significant correlation between EGWG and whether access guidance on nutrition during pregnancy. | **(N=6)**17,21,29,42,49,64 |
| **3** **Policy** |  |  |  |
| C11 Medical insurance policy | C | Two studies (26,46) discovered that pregnant women without medical insurance were more likely to develop EGWG, others suggested that there was no correlation. (59,64,67) | **(N=5)**26,46,59,64,67 |
| **4 Others** |  |  |  |
| C12 Continued community deprivation | S | This factor can reduce the risk of developing EGWG. (33) | **(N=1)**33 |
| C13 Community economic disadvantages | NS | NSED had no relationship with EGWG. (41) | **(N=1)**41 |

EGWG, excessive gestational weight gain

^a^ S: Significant; NS: Non-significant; C: Controversial
