# Supplementary material for: Determinants of excessive gestational weight gain: a systematic review and meta-analysis
Source: Arch Public Health. 2022 May 3;80:129. doi: 10.1186/s13690-022-00864-9 (PMC9066815; doi:10.1186/s13690-022-00864-9)

**Additional file 5** Forest plots of pooled ORs in various factors

- Age (<30 *vs.* ≥30)


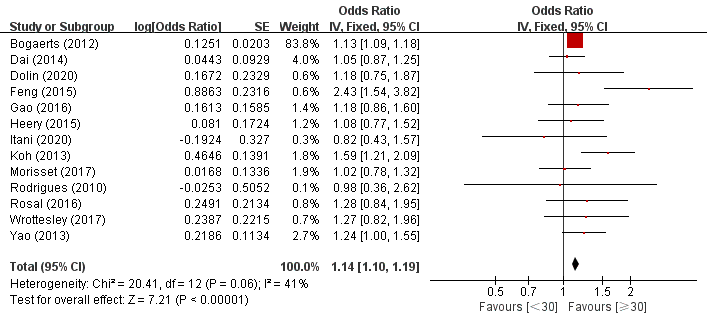


- Employment (No *vs.* Yes)


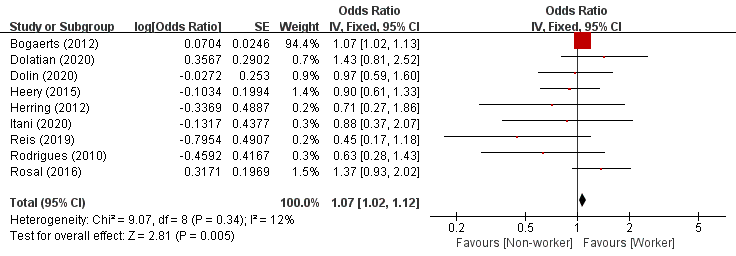


- Pregnancy BMI (Underweight *vs.* Normal)


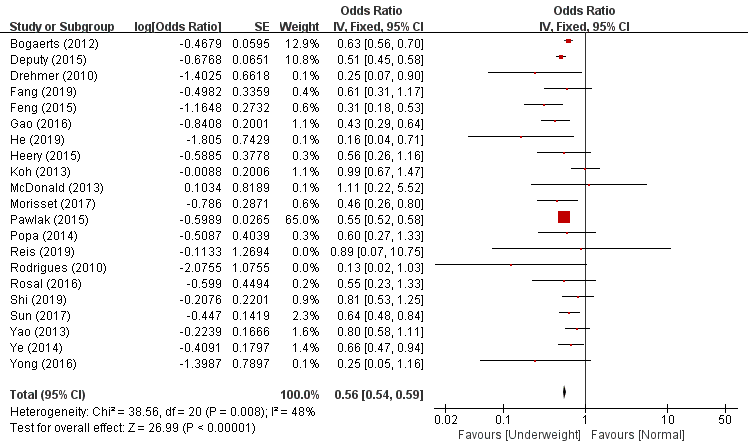


- Pregnancy BMI (Overweight (&obesity) *vs.* Normal)


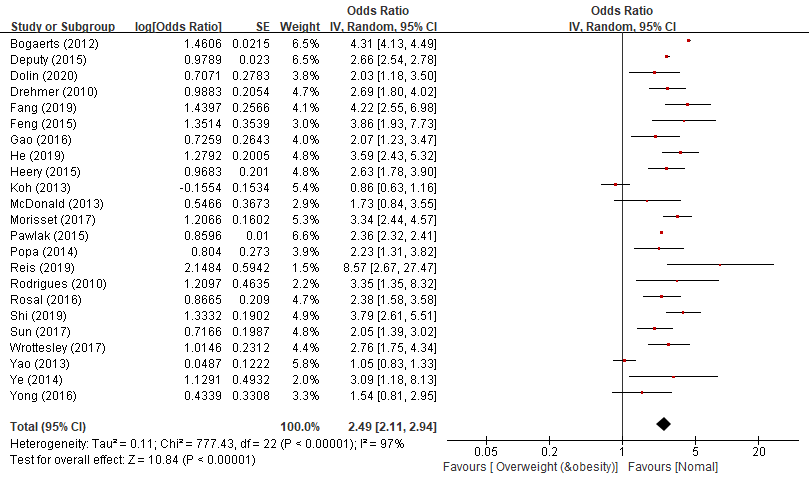


- Parity (Primiparity *vs.* Multiparity)


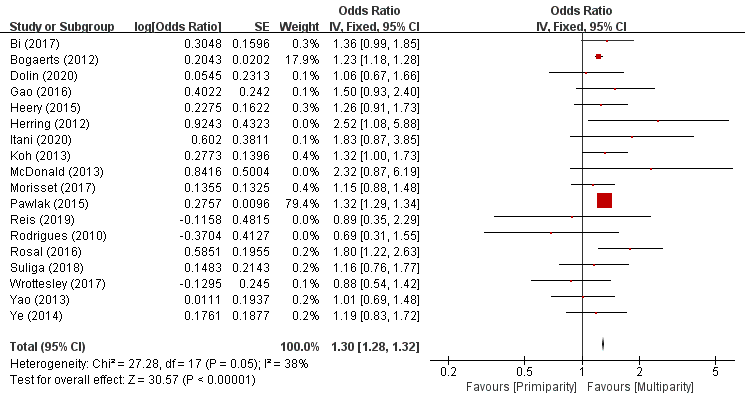


- Planned pregnancy (No *vs.* Yes)


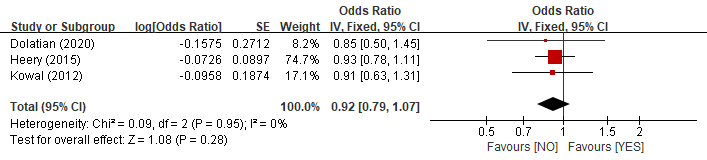


- Smoking (Yes *vs.* No)


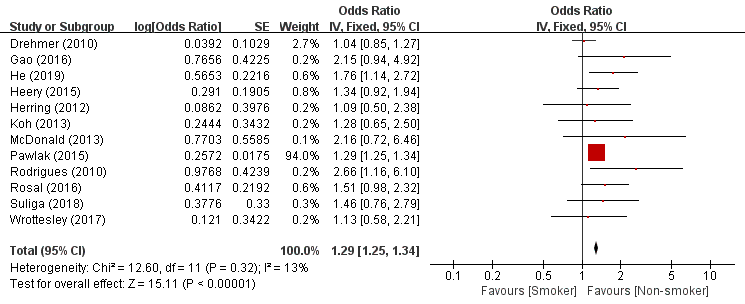


- Alcohol (Yes *vs.* No)


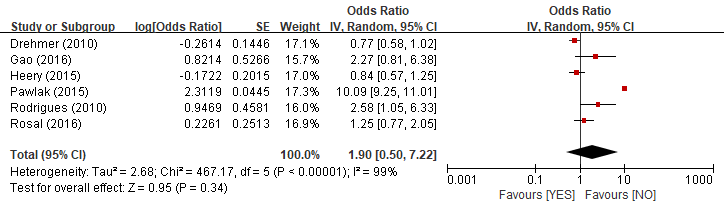


- Prenatal care (Inadequate *vs.* Adequate)


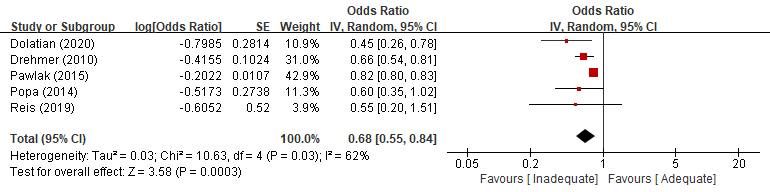


- Marital status (Unmarried (&divorced) *vs.* Married)


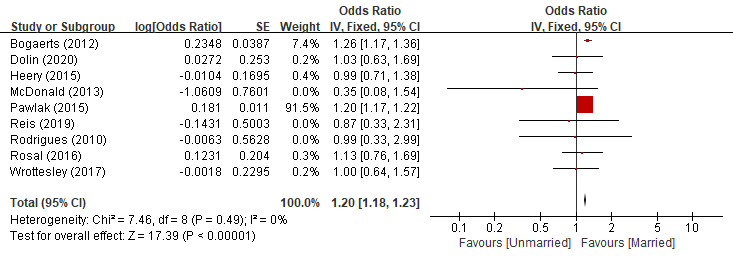


- Food security (No *vs.* Yes)


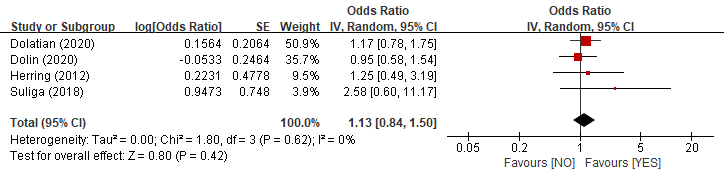


- Nutrition advice or guidance (Not have *vs.* Have)


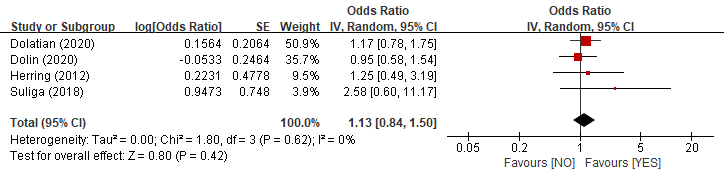

Supplement: Supplementary file 5 — Additional file 5. Forest plots of pooled ORs in various factors. [file 13690_2022_864_MOESM5_ESM.docx]
