# Supplementary material for: Determinants of excessive gestational weight gain: a systematic review and meta-analysis
Source: Arch Public Health. 2022 May 3;80:129. doi: 10.1186/s13690-022-00864-9 (PMC9066815; doi:10.1186/s13690-022-00864-9)
Supplement: Supplementary file 6 — Additional file 6. The results of publication bias. [file 13690_2022_864_MOESM6_ESM.docx]

**Additional file 6** The results of publication bias

Egger’s test of education level (*P* value =.995)

Egger’s test of age (*P* value=.308)

Egger’s test of pre-pregnant overweight (*P* value =.872)

Egger’s test of pre-pregnant underweight (*P* value =.935)

Egger’s test of parity (*P* value =.488)

Egger’s test of smoking (*P* value =.278)

**Figure S1** The results of publication bias by using Egger’s test
